# Long-Term Signs of T Cell and Myeloid Cell Activation After Intestinal Transplantation With Cellular Rejections Contributing to Further Increase of CD16^+^ Cell Subsets

**DOI:** 10.3389/fimmu.2019.00866

**Published:** 2019-05-07

**Authors:** Nadja Stobutzki, Stephan Schlickeiser, Mathias Streitz, Katarina Stanko, Kim-Long Truong, Levent Akyuez, Katrin Vogt, Christine Appelt, Andreas Pascher, Olga Blau, Undine A. Gerlach, Birgit Sawitzki

**Affiliations:** ^1^Institute for Medical Immunology, Charité – Universitätsmedizin Berlin, Corporate Member of Freie Universität Berlin, Humboldt-Universität zu Berlin and Berlin Institute of Health, Berlin, Germany; ^2^Berlin-Brandenburg Center for Regenerative Therapies, Charité – Universitätsmedizin Berlin, Corporate Member of Freie Universität Berlin, Humboldt-Universität zu Berlin and Berlin Institute of Health, Berlin, Germany; ^3^Department of Surgery, Charité – Universitätsmedizin Berlin, Corporate Member of Freie Universität Berlin, Humboldt-Universität zu Berlin and Berlin Institute of Health, Berlin, Germany; ^4^Department for Hematology, Oncology and Tumor Immunology, Charité – Universitätsmedizin Berlin, Corporate Member of Freie Universität Berlin, Humboldt-Universität zu Berlin and Berlin Institute of Health, Berlin, Germany

**Keywords:** intestinal transplantation, T cells, Myeloid cells, flow cytometry, gene expression, cytokines, epigenetics

## Abstract

The intestine mediates a delicate balance between tolerogenic and inflammatory immune responses. The continuous pathogen encounter might also augment immune cell responses contributing to complications observed upon intestinal transplantation (ITx). We thus hypothesized that ITx patients show persistent signs of immune cell activation affecting both the adaptive and innate immune cell compartment. Information on the impact of intestinal grafts on immune cell composition, however, especially in the long-term is sparse. We here assessed activated and differentiated adaptive and innate immune subsets according to time, previous experience of cellular or antibody-mediated rejections or type of transplant after ITx applying multi-parametric flow cytometry, gene expression, serum cytokine and chemokine profiling. ITx patients showed an increase in CD16 expressing monocytes and myeloid dendritic cells (DCs) compared to healthy controls. This was even detectable in patients who were transplanted more than 10 years ago. Also, conventional CD4^+^ and CD8^+^ T cells showed persistent signs of activation counterbalanced by increased activated CCR4^+^ regulatory T cells. Patients with previous cellular rejections had even higher proportions of CD16^+^ monocytes and DCs, whereas transplanting higher donor mass with multi-visceral grafts was associated with increased T cell activation. The persistent inflammation and innate immune cell activation might contribute to unsatisfactory results after ITx.

## Introduction

Intestinal transplantation is an accepted therapeutic option for patients with live-threatening complications upon home parenteral nutrition after intestinal failure (1). The intestine guarantees nutrient absorption but also serves as a protective barrier with contact to commensals and pathogens. Consequently, an efficient communication between local and infiltrating immune cells is needed to maintain a healthy balance between inflammatory responses preventing pathogen invasion and tolerogenic responses against food antigens and commensals (2). Therefore, the small intestine contains various lymphoid structures and thus compared to other transplanted solid organs has a far higher number of donor immune cells (3). Thus, transplantation of an allogeneic intestine represents a major challenge for this tightly controlled balance and ITx patients are prone to more complications than other solid organ transplant recipients. In addition to a higher rate of acute rejection episodes, ITx patients suffer from invasive infections and GvHD (4, 5). Furthermore, the incidence of antibody-mediated humoral rejections is much higher as compared to other transplantations (6–8).

Due to a lack of serum markers indicating an impaired intestinal graft function, diagnosis of rejection episodes requires a combination of clinical, endoscopic examinations, histological assessment of biopsies, and screening for serum donor-specific HLA or non-HLA antibodies (6, 7, 9–13). However, interventions to obtain biopsies are associated with complications and may also trigger injury-related immune reactions. Furthermore, histological discrimination between rejections and viral infections remains challenging warranting the search for novel and particular non-invasive markers.

Indeed, several studies revealed increased numbers or mediators of intragraft and peripheral Th1 and Th17 cells as well as CD8^+^ cytotoxic T cells prior or during rejection (14–21).

Comparatively little is known about the impact on the innate immune cell compartment. Gupta et al. identified a higher myeloid to plasmacytoid DCs balance in pediatric ITx patients with early acute cellular rejection (22).

So far, only individual aspects of the immune system have been studied but no broader assessment of the immune cell composition was performed. In addition, changes in B cell subsets were not analyzed although intestinal grafts contain large numbers of B cells. Also, long-term effects were not studied and no discrimination between cellular and humoral rejections was made.

The intestinal immune compartment especially upon inflammatory challenges is in constant interaction with other immune compartments such as the blood or the liver (23–25). Thus, it appears likely, that in a situation of permanent interaction between self and foreign immune cells major and long-lasting systemic changes in immune cell composition can be observed. Analyzing which subsets are affected will not only provide a more detailed understanding of the interplay of innate and adaptive immune cells and thus immune responses following intestinal transplantation but also give novel insights into the intestine physiology in general.

Consequently, the present study assessed whether upon intestinal transplantation a long-lasting increase in inflammatory differentiated innate and activated adaptive immune cell subsets can be detected. We also investigated immune cell composition in association with organ type or clinical events such as cellular or antibody-mediated rejections. We show that intestinal transplantation is associated with a long-lasting increase in CD16 expressing myeloid mononuclear cells. Conventional T cells showed persistent signs of activation and differentiation, which was counterbalanced by increased levels of activated regulatory T cells. Patients who had experienced cellular rejections showed even higher proportions of CD16^+^ monocytes and myeloid DCs. In contrast, the type of organ and thus donor mass being transplanted was associated with T cell activation.

## Materials and Methods

### Study Populations

Samples from 11 isolated intestinal (i-ITx) and 10 multivisceral transplanted (MVTx) ITx patients (Table 1, between April 2014 and February 2015) and 17 healthy controls of similar age range and gender were collected. We collected three to eight consecutive samples per ITx patient and median results were calculated.

**Table 1:** Characteristics of all isolated (i-ITx) and multivisceral (MVTx) intestinal transplant patients.

**Nr. [Gerlach et al. (7)]**	**Graft**	**Year of Tx**	**Age at Tx years**	**underlying intestinal failure diseases**	**Time post Tx (years)**	**Rejection**	**last time of rejection**	**Pre-Tx HLAabs**	***De novo* HLAabs post-Tx DSA in MFI**	**Average level of immuno-suppressants ng/ml (median [number of tests in time of measurement])**	**Time of measurement**
3	i-ITx	2001	27	Mesenteric infarction	>10	No AR	/	0	/	Tac 4.2 (7); Sir 2.8 (4)	14/06–14/12
5	i-ITx	2001	31	Mesenteric infarction	>10	Humoral/mixed	02/2012	0	DSA A3:1830, A24: 2336, DQ7: 7974	Tac 7.9 (11); Eve 1.0 (10)	14/06–14/12
6	i-ITx	2001	33	Adhesive ileus	>10	Cellular	04/2005	0	/	Tac 4.0 (7); Sir 2.3 (4)	14/07–14/12
9	i-ITx	2002	28	Volvulus	>10	No AR	/	0	/	Tac 3.2 (5); MMF 0.8 (3)	14/07–14/11
11	i-ITx	2003	31	Adhesive ileus	>10	Cellular	11/2009	0	/	Tac 5.2 (8); MMF 3.0 (4)	14/08–14/12
12	MVTx+K	2003	36	Crohn's disease	>10	Humoral/mixed	12/2011	0	NDSA	Tac 5.9 (6); MMF 1.8 (2)	14/06–15/03
16	i-ITx	2007	31	CIPO	4–10	No AR	/	0	/	Tac 5.2 ± 0.5 (9); Sir 1.9 (5)	14/06–14/12
17	MVTx+K	2007	24	Volvulus	4–10	No AR	/	0	/	Tac 4.5 (10); Eve 2.7 (5)	14/06–14/11
18	MVTx	2007	36	Polytrauma	4–10	Humoral/mixed	10/2014	0	DSA B8:997	Tac 6.2 (8); Sir 1.9 (4)	14/07–14/10
21	MVTx	2008	42	Gardner's syndrome	4–10	Humoral/mixed	06/2012	0	NDSA	Tac 5.0 (13); Eve 3.0 (6)	14/07–14/12
22	i-ITx	2008	38	Mesenteric infarction	4–10	Humoral/mixed	03/2013	0	DSA DQ7:6060, DQ8:3938	Tac 4.9 (8); Sir 2.6 (4)	14/06–14/11
23	i-ITx	2009	45	Adhesive ileus	4–10	Humoral/mixed	10/2009	0	DSA A24:1186, DQ7:4278, DQ8: 2457, DR53:4390	Tac 4.1 (8); MMF 0.1 (2)	14/06–14/11
24	i-Itx	2009	44	Mesenteric infarction	4–10	Cellular	10/2009	0	/	Tac 5.9 ± 0.4 (7)	14/06–14/11
26	MVTx	2010	49	Desmoid fibromatosis	4–10	Humoral/mixed	12/2010	NDSA	DSA B60: 2672	Tac 6.8 (11); Eve 4.1 (7)	14/08–14/12
29	MVTx+K	2011	29	Crohn's disease	0–4	No AR	/	NDSA	/	Tac 5.0 (16); Sir 2.7 (5)	14/06–14/12
30	MVTx	2011	52	Adhesive ileus	0–4	cellular	12/2011	NDSA	/	Tac 10.5 (3); Eve 2.8 (3)	14/11
31	MVTx	2012	52	Gardner's syndrome	0–4	Cellular	07/2012		/	Tac 5.6 (15); Eve 3.5 (7)	14/06–14/11
32	MVTx	2013	33	Crohn's disease	0–4	No AR	/		/	Tac 4.5 (3); Eve 2.3 (3)	15/02
33	MVTx	2013	56	Gardner's syndrome	0–4	No AR	/		/	Tac 6.0 (22); MMF 5.0 (4)	14/06–14/12
36	i-ITx	2014	45	Adhesive ileus	0–4	/	/		/	Tac 7.9 (26); Eve 3.2 (13)	14/06–15/03
37	i-ITx	2014	39	Desmoid fibromatosis	0–4	/	/		/	Tac 6.6 (21); Eve 1.8 (6)	14/07–15/02

*Patient 36 and 37 were not included in rejection analysis*.

*i-ITX, isolated intestinal transplantation; MVTX, multivisceral transplantation; K, kidney; Tac, Tacrolimus; Sir, Sirolimus; Eve, Everolimus; MMF, Mycophenolat-Mofetil; DSA, donor-specific anti-HLA antibodies; NDSA, non-donor-specific anti-HLA antibodies; MFI, mean fluorescence intensity*.

Itx patients received induction therapy of thymoglobulin (Thymoglobulin®, Genzyme, Cambridge, Mass., USA; 7.5 mg/kg BW total dose) and one dose of infliximab (Remicade®, Centocor Inc., Essex Pharma GmbH; 5 mg/kg BW). Maintenance immunosuppressive treatment consisted of tacrolimus and rapamycin (sirolimus/everolimus) or MMF (Cellcept®, Hoffmann-LaRoche, Switzerland).

Patients were categorized (i) according to time after transplantation (ITx1 ≥ 10 years, ITx2 = 4–10 years, ITx3 0–4 years) or (ii) according to occurrence of rejections (no AR = no rejection episodes; AR1 = patients with one or several humoral or mixed cellular and humoral rejections; AR2 = patients with one or several cellular rejections). Rejection was defined based on a combination of clinical symptoms and biopsy assessment according to established histological rejection criteria (10, 11). In addition, for diagnosis of humoral rejections assessment of anti-donor HLA antibodies and C4d-staining was performed as previously described (7). Importantly, the tacrolimus trough level was not different between patient groups studied.

All participants gave their written consent to take part in this study authorized by the local ethics committee (Ethikkommission der Charité—Universitätsmedizin Berlin, EA2/044/08 & EA2-020-14).

### Flow Cytometry

Blood samples were stained within 4 h and analyzed by flow cytometry according to the protocol of the ONE-Study Consortium (27, 28). In addition, we included a chemokine receptor panel for categorization of T helper and Treg cell subsets (panel 6, see Supplementary Figure 1 for gating strategy). All fluorochrome-conjugated antibodies used are listed within Supplementary Table 1. In general, 100 μl EDTA blood were directly stained with prepared panel antibody mixes and incubated before lysing erythrocytes with lyse-fix solution composed of Versa Lyse™ and IOTest® Fixative Solution (Beckman Coulter GmbH). For the Treg panel (panel 7) 50 μl EDTA blood were used and additionally stained for intracellular expression of Foxp3 using the PerFix-nc Kit (Beckman Coulter), whereas for the B cell panel (panel 4) 300 μl EDTA blood was first lysed with Red Blood Cell Lysis Solution (Miltenyi Biotec GmbH) prior to antibody staining. The dendritic cell panel 5 was prepared twice and combined after staining. Samples were measured on a 10 color Navios flow cytometer (Beckman Coulter). Calibration with “Flow-Set Pro Beads” and “Flow Check Pro Beads” (both Beckman Coulter) was performed daily.

### T Cell Chimerism Analysis

PBMC were isolated at room temperature by density gradient centrifugation (Biocoll, Biochrom, Berlin, Germany) of heparinized blood diluted 1:2 in Phosphate-Buffered Saline (PBS, Gibco, Thermo Fisher Scientific, Paisley, UK). Cell number was determined using a hemocytometer. Isolated PBMC were cryopreserved until further use. Cryopreserved PBMCs of transplanted patients were first incubated with 3,2 mg/ml human immunoglobulin (Beriglobin, CSL Behring, Germany) for 5 min to block Fc receptors and then stained with anti-TCRαβ-PE, anti-CD4-APC and anti-HLA-DR-ECD (see Supplementary Table 1 for additional information about the antibodies). After washing, cells were stained with 4′,6-diamidino-2-phenylindole (DAPI) and sorted on a FACSAria II (BD Biosciences, Heidelberg, Germany) into DAPI^−^TCRαβ^+^CD4^+^HLA-DR^−^ or –HLA-DR^+^ fractions. Please see Supplementary Figure 4 for gating strategy.

Chimerism analyses were based on the discrimination of donor and recipient alleles on short tandem repeats using PCR with fluorescence-labeled primers. DNA was extracted using a standard DNA extraction method (QIA-Amp; QIAGEN), as recommended by the manufacturer. For quantitative chimerism investigation we used AmpFlSTR® Identifier® PCR Amplification KIT (Applied Biosystems) which contains fluorescent-labeled primer pairs for simultaneous amplification of 16 different loci each. For quantification of chimerism, the areas under the curves were calculated using Genemapper Version 3.7 software (Applied Biosystems). The sensitivity of the method is 1%.

### Real-Time Quantitative Reverse Transcription PCR and TSDR-Demethylation Analysis

Blood samples were collected in Tempus Blood RNA Tubes (Thermo Fisher Scientific, Schwerte, Germany) and RNA was isolated using the MagMAX™ for Stabilized Blood Tubes RNA Isolation Kit (Thermo Fisher Scientific). Up to 1,000 ng RNA were transcribed into cDNA using the QuantiTect Reverse Transcription Kit (Qiagen, Hilden, Germany). Gene expression was measured using TaqMan Gene Expression Assays (Thermo Fisher Scientific, see Supplementary Table 2), microfluidic cards and TaqMan Universal Master Mix (Thermo Fisher Scientific) on the ViiA7 Real Time PCR System (Thermo Fisher Scientific). Reactions were run in duplicates using 384-well microfluific Custom TaqMan® Array Cards. Data were analyzed with ViiA7 Software v 1.2.2. Gene expression was calculated relative to median expression of three reference genes [*Hypoxanthine-guanine phosphoribosyltransferase (HPRT), beta-2-microglobulin (B2M)* and *glyceraldehyde 3-phosphate dehydrogenase (GAPDH)]* using the 2^−ΔΔCt^ method.

Genomic DNA was isolated from EDTA blood using the QIAamp DNA Mini Kit (Qiagen). Up to 2 μg DNA were used for bisulfite treatment (EpiTect, Qiagen). Real-time PCR was done in a final reaction volume of 20 μl with 10 μl FastStart Universal Probe Master (ROX, Roche Diagnostics, Mannheim, Germany), 100 ng Lamda DNA (NEB, Frankfurt a.M., Germany), 5 pmol methylation or non-methylation specific probe, 30 pmol methylation or non-methylation specific primers and at least 15 ng bisulfite-treated DNA or plasmid standard (all Epiontis GmbH, Berlin, Germany). Samples were analyzed in triplicates on an ABI 7500 Cycler (Thermo Fisher Scientific). The percentage of CD4^+^ T cells with demethylated TSDR was calculated by division of non-methylated by total genomic FoxP3 copy-number and normalization to the proportion of total CD3^+^CD4^+^ T cells as determined by flow cytometry.

### Luminex Measurement of Cytokines and Chemokines

Samples were prepared with Milliplex® MAP Kit (Merck KGaA, Darmstadt, Germany) according to manufacturer's protocol. The Bio Plex® 200 Systems (Luminex, Bio-Rad Laboratories GmbH) was validated every 30 days with the Bio-Plex® Validation Kit (Bio-Rad Laboratories GmbH) and was calibrated every day with Bio-Plex® Calibration Kit (Bio-Rad Laboratories GmbH).

### Data Analyses and Statistics

Analysis of flow cytometry data was done with Kaluza version 1.2 (Beckman Coulter). To calculate absolute cell numbers of all reported immune cell subsets, leucocyte cell count was obtained from the clinical chemistry and related to the CD45^+^ count within each panel. The corresponding proportions of all reported immune cell subsets were calculated in Excel. In case of multiple samples from ITx patients a median was calculated. Differences in subset proportions and absolute cell counts between healthy donors and ITx patients as well as according to time post-transplant, rejection or organ type were analyzed with Kruskal-Wallis-Test and a Conover *post-hoc* test. Results were considered as significant when a *p* < 0.05 was reached. *P*-values were not adjusted for multiple testing because of an explorative approach.

R was used for generating a heatmap representation of the mean-centered and sigma-normalized data selected for parameters with a *p* < 0.05 in Kruskal-Wallis-Test, using pairwise euclidean distances and Ward's minimum variance method for hierarchical clustering.

Differences in subset proportions and absolute cell counts between healthy donors and ITx patients as well as according to time post-transplant, rejection or organ type were analyzed with Kruskal-Wallis-Test and a Conover *post-hoc* test. Results were considered as significant when a *p* < 0.05 was reached.

## Results

To investigate whether indeed intestinal transplantation is accompanied by persistent signs of innate and adaptive immune cell activation, we performed multi-parametric profiling of peripheral blood immune cells. From ITx patients three to eight consecutive samples within the observation period were collected and analyzed. The individual immune cell composition was very stable within the observation period (Supplementary Figure 2). From the obtained data median values were calculated.

We assessed the impact of intestinal transplants according to three main hypotheses:

1. The high donor immune cell number and pathogen encounter in ITx grafts triggers persistent immune cell activation resulting in an elevation of activated or inflammatory T and innate immune cell subsets compared to healthy controls.
2. ITx patients show a very slow normalization of immune cell composition normalize over time after transplantation. This was investigated by dividing the transplant patients into three groups: (i) long-term >10 years after transplantation (ITx 1), (ii) mid-term 4–10 years (ITx 2), and (iii) short-term 0–4 years (ITx 3).
3. Previous episodes of acute humoral/mixed rejection (AR 1) or acute cellular rejections (AR 2) are associated with further increase of activated or inflammatory immune cell subsets compared to patients with no rejections (no AR).

### Increase of CD16^+^ Monocytes and DCs in Blood of ITx Patients

**Figure 1:**
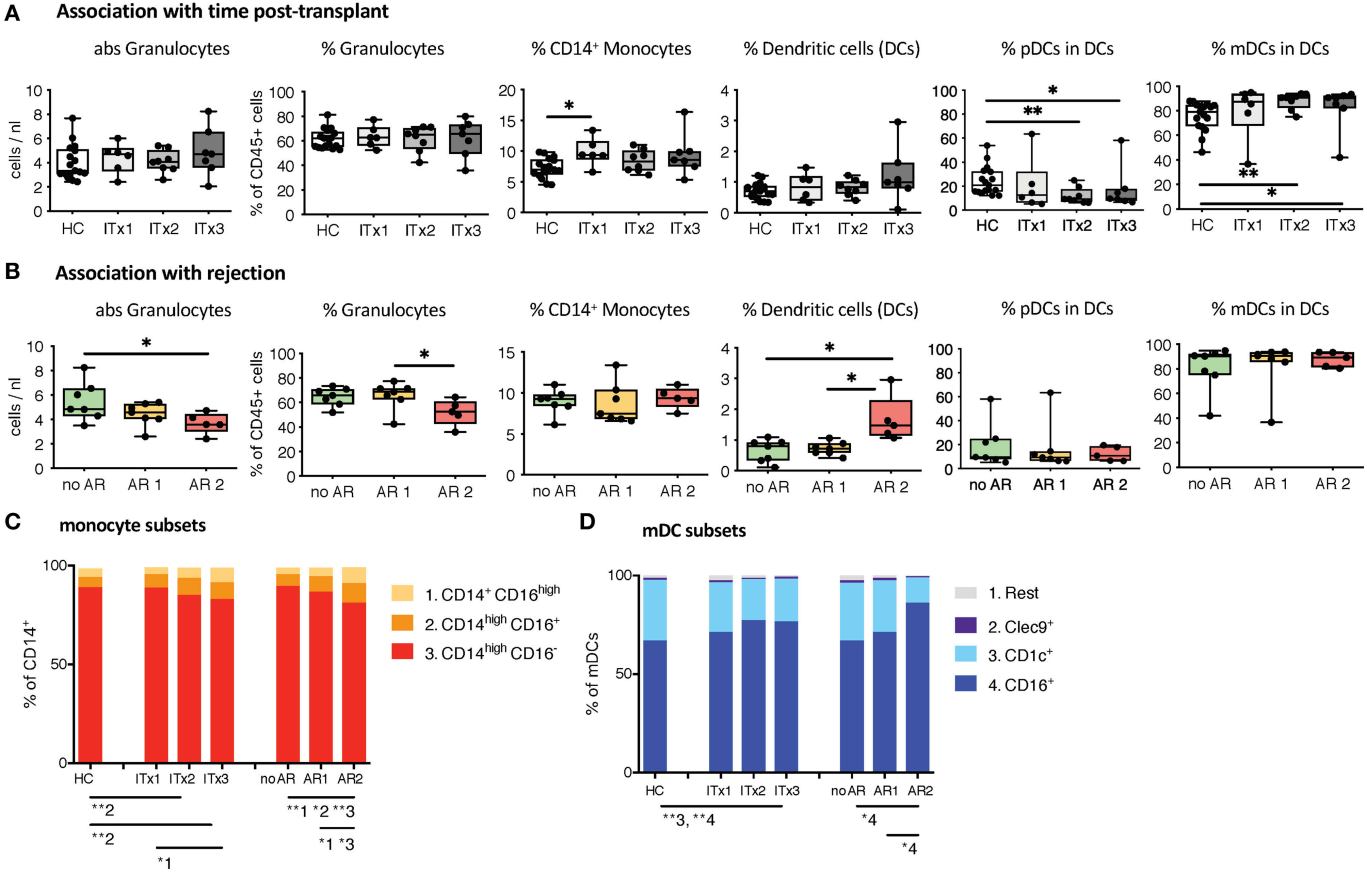
Absolute cell numbers and proportions of indicated innate immune cell subsets in whole blood samples from ITx patients and healthy controls (HCs) were analyzed by flow cytometry. **(A)** Boxplots showing the median and minimum to maximum of results separating the ITx patients according to time after transplantation: 0–4 years (ITx 3, *n* = 7), 4–10 years (ITx 2, *n* = 8), and >10 years (ITx 1, *n* = 6) post-transplantation. The following parameters/cell populations are shown: absolute numbers of granulocytes (identified as CD45^low^SSC^high^) in cells/nl, % of granulocytes of total CD45^+^ leukocytes, % of CD14^+^ monocytes of total CD45^+^ leukocytes, % LIN^−^HLA-DR^+^ dendritic cells of total CD45^+^ leukocytes, % CD11c^−^CD123^+^ plasmacytoid dendritic cells of dendritic cells, and CD11c^+^ myeloid dendritic cells of dendritic cells. **(B)** Boxplots showing the median and minimum to maximum of results separating the ITx patients according to clinical rejection episodes: no rejection (no AR, *n* = 7), acute humoral/mixed rejection (AR 1, *n* = 7), and acute cellular rejection (AR 2, *n* = 5). **(C)** Mean proportions of monocyte subsets (1 = CD14^+^CD16^high^, 2 = CD14^high^CD16^+^, 3 = CD14^high^CD16^−^) in ITx patients separated according to time after transplantation or occurrence of rejections. **(D)** Mean proportions of myeloid dendritic cell subsets (1 = rest, 2 = Clec9^+^, 3 = CD1c^+^, 4 = CD16^+^) in ITx patients separated according to time after transplantation or occurrence of rejections. Statistical analysis was done using a Kruskal-Wallis-Test and Conover *post-hoc* test. *p < 0.05, **p < 0.01.

**Figure 2:**
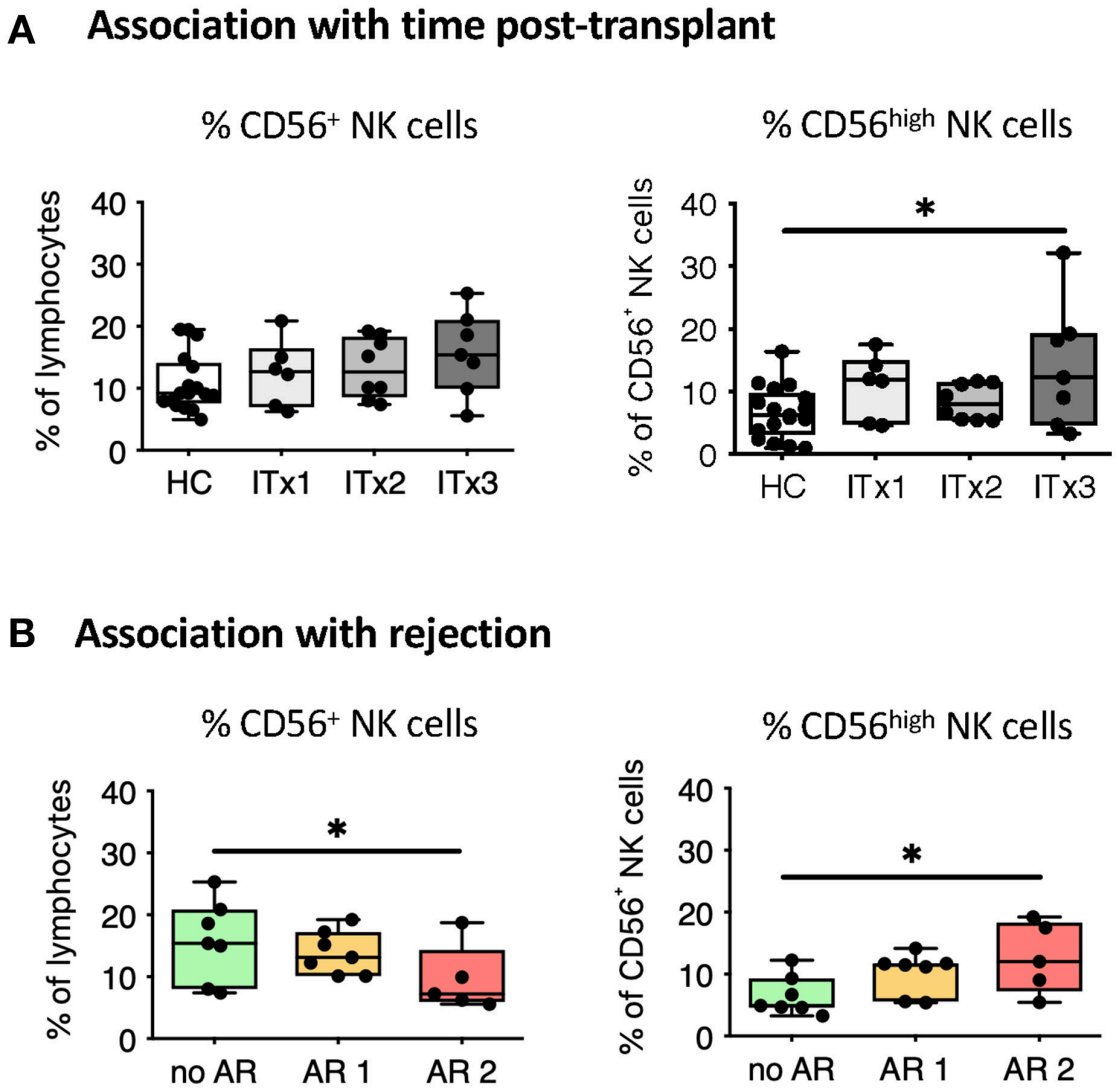
Comparative analysis of NK cell subsets. Boxplots showing the median and minimum to maximum of proportions of total NK cells **(A,B)**, CD56^high^ NK cells **(A,B)** in whole blood samples from patients according to time post-transplant (A: ITx = 0–4 years, *n* = 7; ITx = 4–10 years, *n* = 8; ITx > 10 years, *n* = 6) or occurrence of rejections (**B**: no AR = no rejection, *n* = 7; AR1 = humoral/mixed rejection, *n* = 7; and AR2 = acute cellular rejection, *n* = 5) and healthy controls (HCs). Statistical analysis was done using a Kruskal-Wallis-Test and Conover *post-hoc* test. *p < 0.05.

First, we investigated differences in proportions and absolute numbers of innate immune cells including granulocytes, monocytes, DCs, NK cells, and their major subsets (Figures 1, 2 and Supplementary Tables 3, 4). We could not detect differences in granulocytes between samples from healthy controls and transplant patients (Figure 1A). However, there was a significant decrease in absolute granulocyte numbers in samples of patients who had experienced cellular rejections (AR2, Figure 1B and Supplementary Tables 3, 4). Total monocytes were increased in long-term transplant patients (Figure 1A). The proportions and absolute numbers of the CD14^high^CD16^+^ subset were increased in patient samples especially early after transplantation and proportions of both CD16 expressing monocytes subsets were higher in patients experiencing cellular rejections (Figure 1C and Supplementary Tables 3, 4).

Although samples from transplant patients did not contain more DCs, they were higher in patients with previous cellular rejections (Figure 1B). We also observed an altered balance between plasmacytoid and myeloid DCs in patients (Figure 1A) resulting from an increase in the CD16^+^ subset (Figure 1D). This increase of CD16^+^ myeloid DCs was especially prominent for patients who had cellular rejections.

There was a trend toward higher proportions of NK cells early after transplantation (ITx3, Figure 2A), mainly due to an expansion of CD56^high^ NK cells normalizing 10 years after transplantation (ITx1). Increase of total CD56^+^ NK cells was seen in stable patients without rejection episodes (no AR, Figure 2B), who had similarly low proportions of CD56^high^ NK cells compared to healthy controls.

Altogether, ITx patients have major alterations in their systemic innate immune compartment either occurring independently from clinical events or associated with rejections. Changes related to rejections were independent of time since last rejection episode (data not shown). The organ type being transplanted did no influence composition of innate immune cell subsets (Supplementary Figure 3A).

### Increased Proportions of Activated and Differentiated Conventional Recipient T Cell Subsets Also in Patients With No Rejections

**Figure 3:**
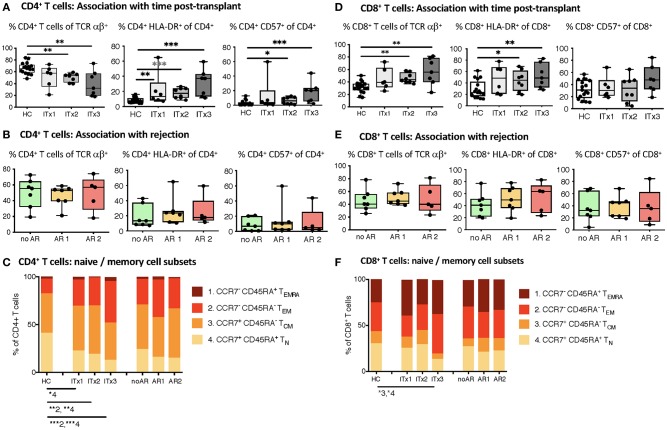
Comparative analysis of αβ T cell memory differentiation and expression of activation markers measured by flow cytometry. Shown are proportions as boxplots (median and minimum to maximum) of total, activated HLA-DR or CD57 expressing CD4^+^ **(A,B)** and CD8^+^ αβ T cells **(D,E)** as well as their CD45RA^+^CCR7^+^ naïve (1, T_N_), CD45RA^−^CCR7^−^ central memory (2, T_CM_), CD45RA^−^CCR7^−^ effector memory (3, T_EM_), and CD45RA^+^CCR7^−^ terminal differentiated effector memory (4, T_EMRA_) subpopulations **(C,F)** measured in whole blood samples from intestinal transplant patients separated according to time post-transplant (ITx = 0–4 years, *n* = 7; ITx=4–10 years, *n* = 8; ITx > 10 years, *n* = 6) or occurrence of rejections (no AR = no rejection, *n* = 7; AR1 = acute humoral/mixed rejection, *n* = 7; and AR2 = acute cellular rejection, *n* = 5) and healthy controls. Statistical analysis was done using a Kruskal-Wallis-Test and Conover *post-hoc* test. *p < 0.05, **p < 0.01, ***p < 0.001.

**Figure 4:**
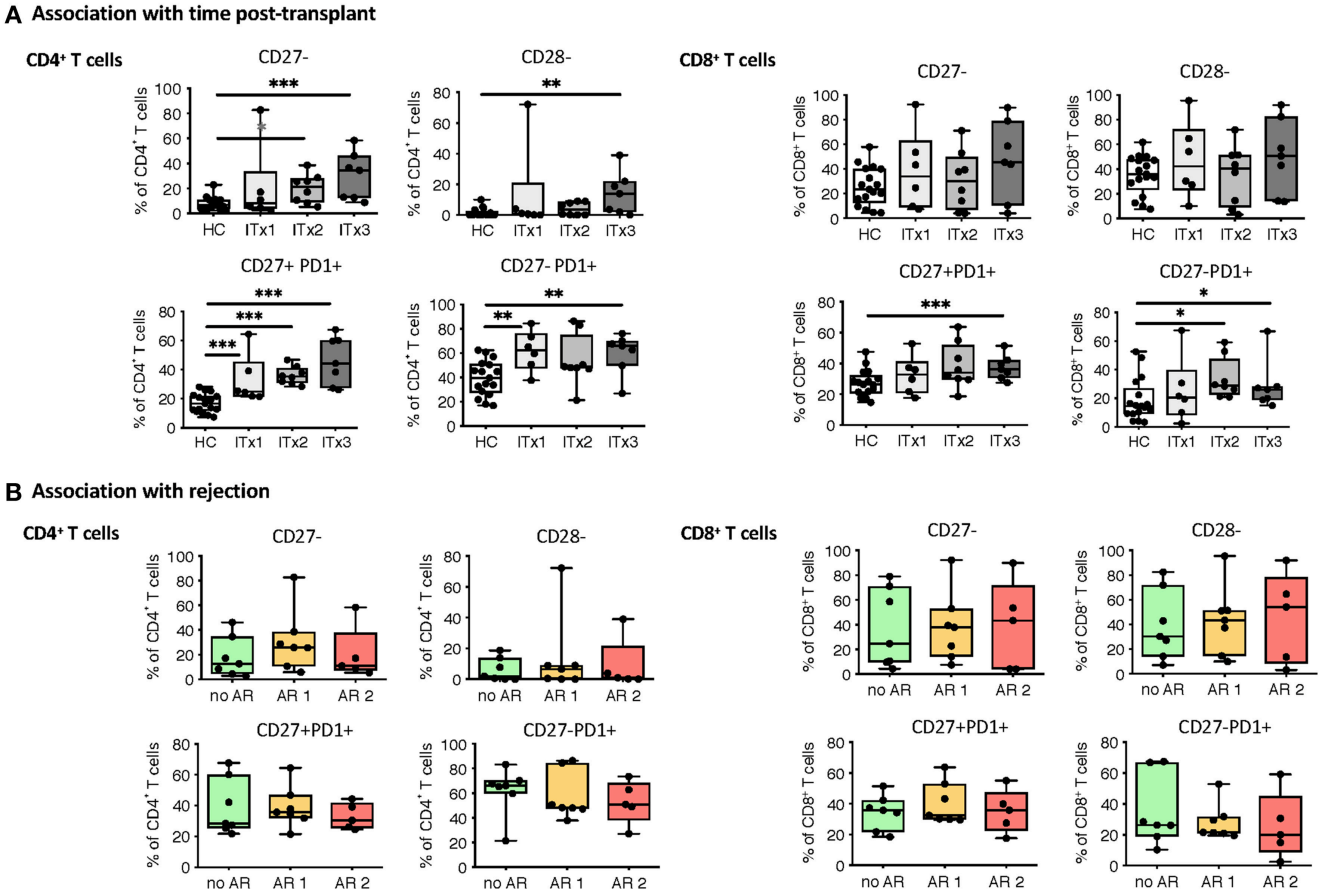
Analysis of T cell senescence and exhaustion markers. Boxplots of proportions (median and minimum to maximum) of CD27^−^, CD28^−^ as well as PD1 expressing subpopulations of CD4^+^ and CD8^+^ T cells in whole blood patient samples and healthy control samples were determined by flow cytometry and plotted according to time post-transplant (**A**: ITx = 0–4 years, *n* = 7; ITx = 4–10 years, *n* = 8; ITx > 10 years, *n* = 6) or occurrence of rejection episodes (**B**: no AR = no rejection, *n* = 7; AR1 = acute humoral/mixed rejection, *n* = 7; and AR2 = acute cellular rejection, *n* = 5). Statistical analysis was done using a Kruskal-Wallis-Test and Conover *post-hoc* test. *p < 0.05, **p < 0.01, ***p < 0.001.

T cells are known to play a major role in controlling anti-donor immune responses and eliciting rejections. Therefore, changes in composition of helper and cytotoxic T cells and their activated subsets were assessed. CD4^+^ (TCRαβ^+^) T helper cell proportions and absolute numbers were significantly lower in transplant patient samples especially early after transplantation (ITx2 & 3, Figure 3A and Supplementary Tables 3, 4). This was independent of rejections (Figure 3B). Furthermore, a large proportion of the T helper cells showed signs of acute and chronic activation e.g., HLA-DR and CD57 expression, respectively (Figure 3A). Consistently, transplant patients showed a reduction of naïve T cells (Figure 3C) and strong increase of CD27^−^ late stage effector like T cells (Figure 4A). Although these changes were especially apparent early after transplantation, they were still detectable in patients who had been transplanted more than 10 years ago (ITx1). Similar observations were made for CD8^+^ cytotoxic T cell subsets (Figures 3D, 4A). Much to our surprise, the increase in activated and differentiated T cell subsets was not significantly higher in patients who had experienced rejections Figures 3B,C,E,F and 4B. However, we detected higher proportions of HLA-DR^+^, CD57^+^, or CD27^−^ T cells in patients who received a multi-visceral transplant (Supplementary Figure 3B). It has been previously shown that ITx patients especially early after transplantation display T cell donor chimerism (29). Therefore, we investigated whether the activated T cells are of donor or recipient origin. We have FACS-sorted CD4^+^ T cells from frozen PBMCs of nine different ITx patients (three of each ITx group, mixed balance of isolated and multivisceral transplanted patients) into HLA-DR^+^ and HLA-DR^−^ proportions (see also new Supplementary Figure 4). Afterwards, DNA was isolated and the degree of chimerism determined by PCR utilizing fluorescence-labeled primers which discriminate between donor and recipient alleles. Interestingly, in none of the samples, neither in HLA-DR^+^ activated nor in HLA-DR^−^ non-activated cells, we could detect donor alleles (0% donor chimerism in all samples).

Thus, CD4^+^ and CD8^+^ systemic recipient T cell compartment of ITx patients is heavily altered showing signs of constant T cell activation mostly dependent on donor mass being transferred.

### Increase in Proportions of Chemokine Receptor Expressing Subsets of Conventional T Helper and Regulatory T Cells

**Figure 5:**
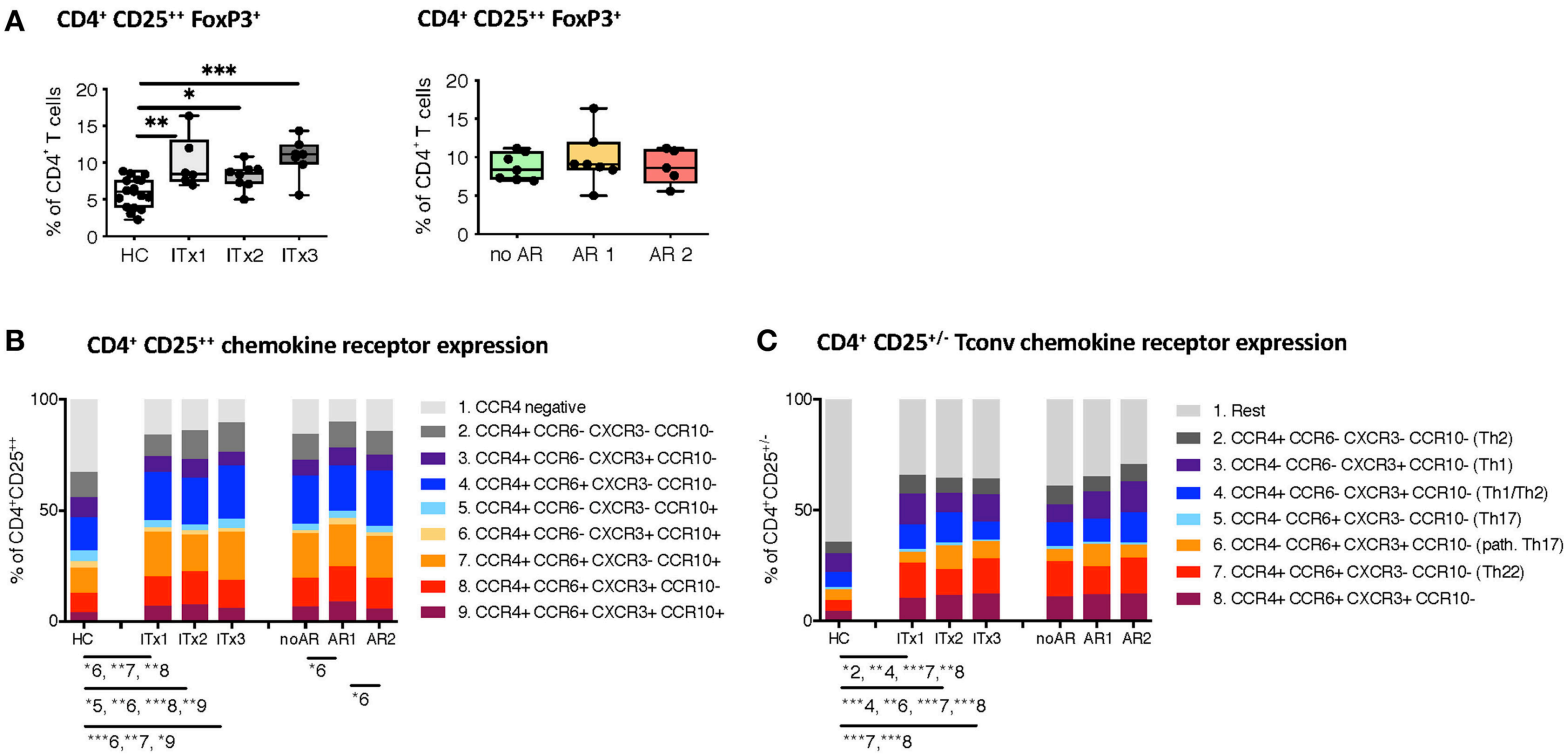
Regulatory T cells and conventional T helper subsets. **(A)** Proportions shown as boxplots (median and minimum to maximum) of total CD4^+^CD25^++^Foxp3^+^ regulatory T cells in whole blood samples from patients and healthy controls displayed according to time after transplantation (left: ITx = 0–4 years, *n* = 7; ITx = 4–10 years, *n* = 8; ITx > 10 years, *n* = 6) or occurrence of rejections (right: no AR = no rejection, *n* = 7; AR1 = acute humoral/mixed rejection, *n* = 7; and AR2 = acute cellular rejection, *n* = 5). **(B)** Overview on proportions of CCR4^+^ activated memory regulatory T cells and their co-expression of CCR6, CXCR3, or CCR10 within whole blood patient samples and healthy control samples. **(C)** Proportions of CD25^−/+^ conventional T cells expressing CCR4, CCR6, or CXCR3 resembling a Th2, Th1, TH1/Th2, Th17, pathogenic Th17 or Th22-like phenotype in patient and healthy control samples. Statistical analysis was done using a Kruskal-Wallis-Test and Conover *post-hoc* test. *p < 0.05, **p < 0.01, ***p < 0.001.

With intestinal transplantation representing a major inflammatory event it might trigger upregulation of chemokine receptor expression enhancing the homing potential of circulating T cells. Investigation of chemokine receptor expression was done for regulatory and conventional CD4^+^ T cells. Proportions but not absolute numbers of CD4^+^CD25^++^Foxp3^+^ regulatory T cells (Tregs) were significantly higher in transplant patients even 10 years after transplantation compared to healthy controls (Figure 5A and Supplementary Tables 3, 4). In accordance, the percentage of CD4^+^ T cells with a demethylation of the TSDR determining stable Foxp3 expressing Tregs were higher in ITx patients but independent of rejections (Supplementary Figures 5A,C). However, we did not observe differences in Foxp3 mRNA expression (Supplementary Figures 5B,D).

Nearly all of the systemic Tregs showed an activated CCR4 expressing phenotype (30). Furthermore, the majority displayed CCR6 co-expression (Figure 5B).

We also assessed proportions and absolute numbers of conventional T cells (CD25^−/+^) expressing a Th1-like (CCR4^−^CCR6^−^CXCR3^+^CCR10^−^), Th2-like (CCR4^+^CCR6^−^CXCR3^−^CCR10^−^), Th17-like (CCR4^−^CCR6^+^CXCR3^−^CCR10^−^), Th22-like (CCR4^+^CCR6^+^CXCR3^−^CCR10^−^), mixed Th1/Th2-like (CCR4^+^CCR6^−^CXCR3^+^CCR10^−^), and pathogenic Th17-like (CCR4^−^CCR6^+^CXCR3^+^CCR10^−^) chemokine receptor expression profile (Figure 5C and Supplementary Tables 3, 4) (31, 32). CCR10 expressing T cells represented only a minor fraction of conventional CD4^+^ T cells. Therefore, we did not capture CCR10 expressing subsets. We did not detect differences in Th1-like T helper cells neither when comparing transplant patients and healthy controls nor according to rejections. Similarly, Th17-like cells occurred at very low numbers and were not different between groups. In contrast, Th2-like cells seem to accumulate over time in ITx patients (ITx1). We also observed a significant increase in proportions of populations expressing several chemokine receptors such as Th22-like, pathogenic Th17-like or Th1/Th2-like cells in transplant patients (Figure 5C).

Thus, ITx patients are characterized by higher proportions of chemokine receptor expressing regulatory and conventional CD4^+^ T cells regardless whether they experienced rejections or not.

### High Systemic Chemokine and Cytokine Levels Even in Stable Transplant Patients

As we did detect increased proportions of chemokine receptor expressing T helper cells in ITx patients, we also investigated serum chemokine and cytokine concentrations.

**Figure 6:**
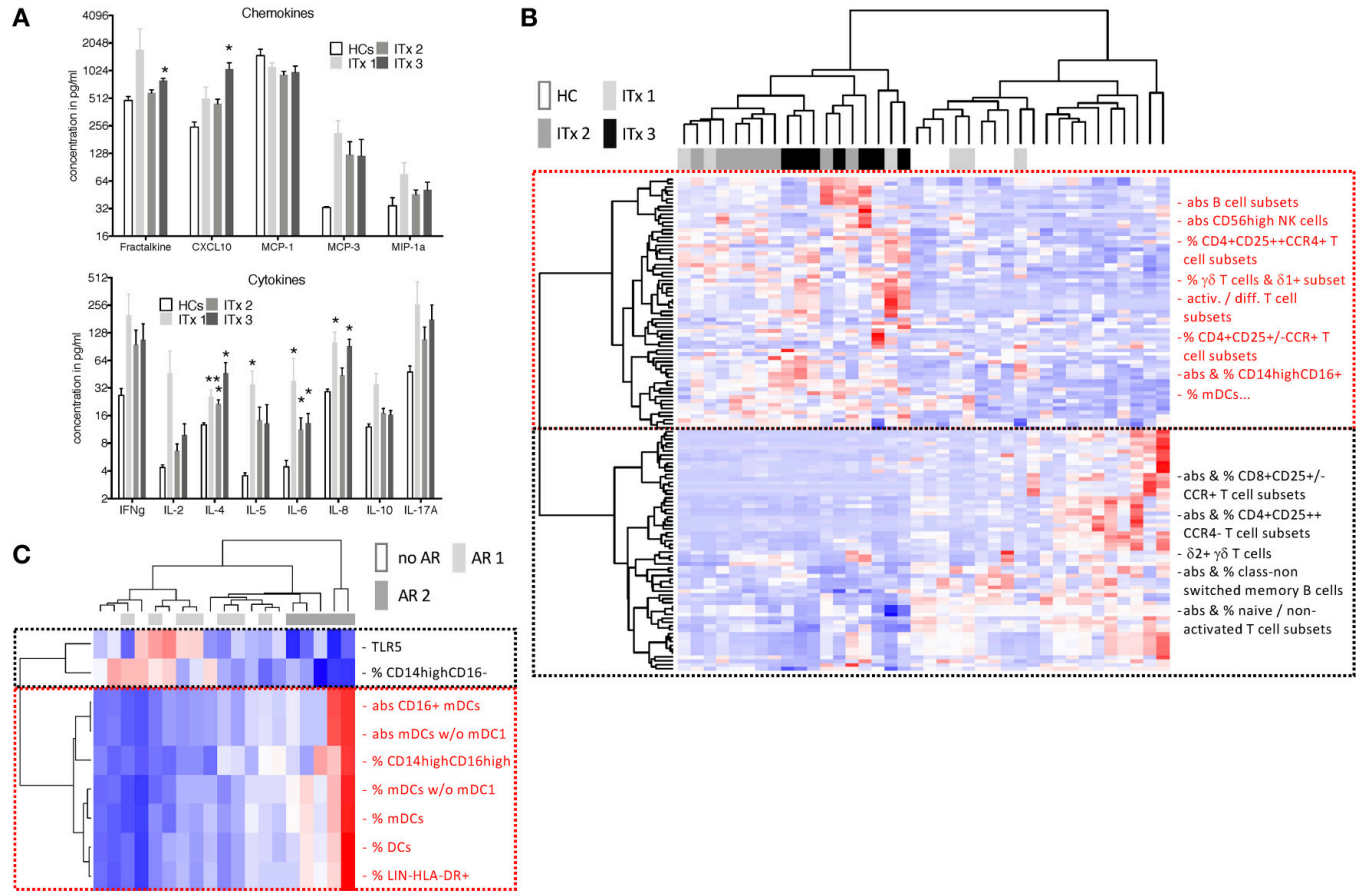
Serum cytokines, chemokines as well as heatmaps summarizing differences according to time and rejection. **(A)** Chemokine and cytokine levels in serum samples of transplant patients (Tx, *n* = 18) and healthy controls (HCs, *n* = 4) were measured using the luminex technology. Transplant patients were categorized according to time after transplantation (ITx1: *n* = 6, ITx2: *n* = 8, ITx3: *n* = 3). Statistical analysis was done using a Kruskal-Wallis-Test and Conover *post-hoc* test. *p < 0.05 **p < 0.01. **(B,C)** heatmaps summarizing parameters that differ according to time and rejection (euclidean distance, unadjusted *p* < 0.01). Blue boxes = low values, red boxes = high values. **(B)** Comparison of ITx patients with HCs. ITx were divided according to time since Tx in long- (ITx 1: >10 years), mid- (ITx 2: 4–10 years), and short-term (ITx 3: 0–4 years). Clusters (dashed boxes) are comprised mainly of activated/differentiated/pathogenic (red) and naive/non-activated (black) immune subsets. **(C)** Analysis of acute cellular (AR 2), humoral/mixed (AR 1) and no rejection (no AR) in ITx patients. Complete list of all parameters that differ according to time and rejection after transplantation are given in Supplementary Table 6.

Concentrations of chemokines attracting T cells and macrophages such as CXC3CL1, CXCL10, CCL2, CCL7, CCL3 as well as cytokines released by T helper subsets such as Interleukin (IL)-2, IL-4, IL-5, IL-10, IL-17A, or interferon gamma (IFN-γ) were determined. Serum samples from patients early after transplantation (ITx3) contained more CXC3CL1 and CXCL10 compared to those from healthy controls (Figure 6A). Furthermore, cytokines typically produced by Th2 cells such as IL-4 and IL-5 were significantly elevated also in long-term transplant patients (Figure 6A). Although Th1 (IL-2, IFN-g) and Th17 cytokines (IL-17A) showed a tendency to be increased in serum samples of transplant patients, this did not reach significance. We only detected a tendency toward higher fractalkine, IL-2, and IL-4 concentrations in serum samples of patients with cellular rejections (Supplementary Figures 6A,B).

Altogether, we detected high systemic T cell attracting chemokines and Th2 cytokines in ITx patients even long-term after transplantation and independent of rejections.

### Cluster Analysis of Parameters Contributing to Patient Group Separation

To obtain a better overview of parameters contributing to patient group separation according to time and rejection after intestinal transplantation we performed a cluster analysis of all parameters which had an unadjusted *p* < 0.05 comparing healthy controls to early (ITx3), mid-term (ITx2), and long-term (ITx1) transplant patients (Figure 6B) or comparing patients with regard to occurrence of rejections (Figure 6C). We also included qRT-PCR results of gene markers previously described to be highly expressed in operational tolerant or acutely/chronically rejecting kidney transplant recipients (Supplementary Table 5).

The heatmap shown in Figure 6B confirms, that nearly all ITx patients are distinct to healthy controls. Populations contributing mostly to the separation and being higher in ITx patients were belonging to the B cell lineage (e.g., total B cells, naïve B cells). Indeed, gene markers known to be highly expressed by transitional and naïve B cells such as *Membrane Spanning 4-Domains A1* (*MS4A1*), *CD79B*, and *T-Cell Leukemia/Lymphoma 1A* (*TCL1A*) were enriched in samples from ITx patients. Further populations and marker contributing to the separation were CD16^+^ monocytes, CD56^high^ NK cells, γδ^+^ T cells, memory/activated/chemokine receptor expressing CD4^+^ T cell subsets as well as IL-4.

In contrast, CD16^−^ monocytes, CD56^dim^ NK cells as well as naïve/non-activated CD4^+^ and CD8^+^ T cell subsets were high in healthy controls. A complete list of all cell populations or gene markers contributing to the separation by being either higher (upper main cluster) or reduced (lower main cluster) ITx patients is provided within Supplementary Table 6.

As shown in Figure 6C significant differences in CD16 expressing monocytes and DCs as well as TLR5 expression allowed separation of patients who never experienced rejection episodes (no AR) or humoral rejections (AR1) from all patients who had cellular rejections (AR2). However, separation of patients with humoral rejections (AR1) from stable patients (no AR) was not possible.

## Discussion

We have performed comprehensive assessment of the systemic innate and adaptive immune system according to time, rejections or organ type after intestinal transplantation. Our results revealed that ITx patient samples contained more CD16 expressing monocytes and myeloid DCs independent of rejections. This was detectable even 10 years after transplantation. Similarly, we observed enhanced proportions of activated conventional recipient T cells which showed a broad T helper cell chemokine expression profile associated with constant high serum Th1, Th2, and Th17 cytokine levels in comparison to healthy control samples. This increase in activated T helper cells was associated with the organ type being transplanted and counterbalanced by enhanced proportions of activated regulatory T cells. Furthermore, the persistent signs of T cell activation were not related to pre-transplant sensitization as none of the patients had DSA prior to transplantation (Table 1).

Cellular rejections induced an even more dramatic increase in total DCs, CD16 expressing monocytes and DCs as well as CD56^high^ NK cells whereas proportions of total NK cells were reduced. Together with reduced peripheral TLR5 expression this pattern enabled clear separation of patients with previous cellular rejection from stable patients and patients with humoral rejections. Importantly this was not related to differences in IS as tacrolimus trough level were not different between all three patient groups.

Previous reports revealed increased proportions of myeloid cells, monocytes and DCs, in pediatric ITx patients experiencing acute rejections (22, 33). In our cohort CD16 expressing monocytes and myeloid DCs were also highest in patients with cellular rejections. Elevated proportions of CD16 expressing monocytes have been observed in patients with ongoing inflammation such as chronic kidney disease patients (CKD) and are discussed to reflect endothelial damage (34).

In previous studies enhanced frequencies of donor-reactive CD154^+^ activated memory T cells in rejecting patients have been observed (14, 21). Our analysis revealed a general and persistent high T cell activation, T helper cell differentiation and memory T cell formation with no obvious differences according to previous experience cellular or humoral rejection episodes but rather donor mass being transplanted. The effect of antigen mass is in accordance with other previous investigations (35). Most of the samples from patients experiencing cellular or humoral rejections were collected months or years after the last rejection episode, which might explain the discrepancies. Also, our main aim was not to identify predictive biomarkers of or diagnose rejection, but to determine how intestinal transplantation including accompanying clinical events such as rejections affects systemic immune cell composition and activation. Surprisingly and in contrast to previous studies that revealed a high degree of donor T cell chimerism especially early after transplantation and in patients receiving multi-visceral transplants (29, 36, 37), we could not detect donor alleles in peripheral blood CD4^+^ T helper cells, neither in the activated HLA-DR^+^ nor in the non-activated HLA-DR^−^ subpopulation. Thus, the constant high proportions of activated T cells are not a sign of persistent chimerism.

Increased pDCs have been associated with development of tolerance upon e.g., liver transplantation (38, 39). Thus, the persistently low pDC/mDC ratio and high proportion of the inflammatory CD16 expressing monocytes and DCs detected in our patient cohort might be an indication of constant inflammation.

We detected a significantly decreased absolute number and proportions of granulocytes in samples of patients with a previous cellular rejection. Indeed, intragraft sequestration of neutrophils has been described during rejection but for both cellular (26) and antibody-mediated rejection (40, 41). As the performed blood sample analysis happened long-time after rejection, the sequestration might continue even in the absence of clinical rejection symptoms.

Furthermore, composition of NK subsets was more severely altered in patients who had experienced rejection episodes. Patients with cellular showed lower total NK cells, and a higher proportion displayed a more differentiated CD56^high^ phenotype. This might reflect their rejection-dependent activation and intragraft accumulation. However, this is contrary to previous reports which showed reduced circulating NK cells and elevated proportions of CD56^bright^ NK cells in renal allograft recipients with DSA and non-DSA anti-HLA mAbs or NK-cell related transcripts in AMR biopsies (42–44).

Regardless of the time post-transplant or occurrence of rejections ITx patients displayed increased systemic proportions of αβ CD4^+^CD25^++^Foxp3^+^ regulatory T cells. The majority of the Tregs had a CCR4^+^ activated memory phenotype with co-expression of predominantly CCR6. Our results question the conclusions drawn from a recently published study in which high percentages of Tregs upon intestinal transplantation were ascribed to a special immunomodulatory protocol and associated with improved long-term graft function (45). In this study, ITx patients receiving the immunomodulatory protocol were compared to kidney transplant patient groups and healthy controls but not to ITx patients on other immunosuppressive medications. Thus, high proportions of CCR6^+^ Tregs might rather indicate permanent recruitment to counterbalance ongoing immune cell activation following intestinal transplantation.

Our study has some limitations: the patient cohort was relatively small and no intragraft sampling was performed. In addition, given the high mortality rate of intestinal transplant patients within the first years after transplantation, the long-term groups (ITx1 and ITx2) have a bias toward survivors. Therefore, it is even more surprising to observe these alterations in immune cell composition long term after transplantation in the surviving patients.

Furthermore, this is the first comprehensive assessment of time-dependent and rejection-dependent alterations of systemic immune cell composition. Our findings clearly reveal persistent inflammatory responses and activation of immune cells upon intestinal transplantation potentially contributing to unsatisfactory long-term results compared to other solid organ transplants. In future, it will be important to perform comparative investigations in other solid organ graft recipients, but this was clearly beyond the scope of our study.

## Ethics Statement

All participants gave their written consent to take part in this study authorized by the local ethics committee (Ethikkommission der Charité—Universitä tsmedizin Berlin, EA2/044/08 & EA2-020-14).

## Author Contributions

NS and KS acquisition and analysis of data, drafting of manuscript. SS data interpretation and statistical analysis. MS analysis of data. OB acquisition and analysis of data and K-LT critical revision of the manuscript. LA, KV, and CA technical support and acquisition of data. AP and UG material support and critical revision of the manuscript. BS study concept and design, obtained funding, and drafting of manuscript.

### Conflict of Interest Statement

The authors declare that the research was conducted in the absence of any commercial or financial relationships that could be construed as a potential conflict of interest.

## Abbreviations

DCs: dendritics cells
EDTA: ethylenediaminetetraacetate
Foxp3: forkhead box P3
GvHD: graft vs. host disease
HLA: human leukocyte antigen
ITx: intestinal transplantation
MMF: mycophenolate mofetil
NK: natural killer cells
Th1: T helper 1 cells
Th2: T helper 2 c ells
Th17: T helper 17 cells
Th22: T helper 22 cells
Treg: T regulatory cells
TSDR: Treg specific demethylation region.
