# Supplementary material for: Long-Term Signs of T Cell and Myeloid Cell Activation After Intestinal Transplantation With Cellular Rejections Contributing to Further Increase of CD16+ Cell Subsets
Source: Front Immunol. 2019 May 7;10:866. doi: 10.3389/fimmu.2019.00866 (PMC6514047; doi:10.3389/fimmu.2019.00866)
Supplement: Supplementary file 1 [file Table_1.pdf]

Suppl. table 1: Panel set-up used for multi-parametric flow cytometry

Panel	Target antigen conjugate	Company
1	CD45-KrOrange	Beckman Coulter (B36294)
	CD3-APC-A750	Beckman Coulter (A94680)
	CD4-APC	Beckman Coulter (IM2468)
	CD8-APC-A700	Beckman Coulter (CD)
	CD14-PC7	Beckman Coulter (A22331)
	CD16-FITC	Beckman Coulter (IM0814U)
	CD19-ECD	Beckman Coulter (A07770)
	CD56-PE	Beckman Coulter (A07788)
2	CD45-KrOrange	Beckman Coulter (B36294)
	CD3-APC-A750	Beckman Coulter (A94680)
	CD4-APC	Beckman Coulter (IM2468)
	CD8-APC-A700	Beckman Coulter (CD)
	TCRab-PE	Beckman Coulter (A39499)
	TCRgd-FITC	Beckman Coulter (IM1571U)
	TCR-Vd1-PC7	Beckman Coulter (CD)
	TCR-Vd2-PacBlue	Beckman Coulter (CD)
	HLA-DR-ECD	Beckman Coulter (IM3636)
3	CD45-KrOrange	Beckman Coulter (B36294)
	CD3-APC-A750	Beckman Coulter (A94680)
	CD4-APC	Beckman Coulter (IM2468)
	CD8-APC-A700	Beckman Coulter (CD)
	CD27-PC7	Beckman Coulter (A54823)
	CD28-ECD	Beckman Coulter (6607111)
	CD57-PacBlue	Beckman Coulter (A74779)
	PD1-PC5.5	Beckman Coulter (B36123)
	CD45RA-FITC	Beckman Coulter (6603904)
	CCR7-PE	Beckman Coulter (B30632)
4	CD45-KrOrange	Beckman Coulter (B36294)
	CD19-ECD	Beckman Coulter (A07770)
	CD21-PE	Beckman Coulter (A32536)
	CD24-APC	Beckman Coulter (A87785)
	CD27-PC7	Beckman Coulter (A54823)
	CD38-APC-A700	Beckman Coulter (A86049)
	IgD-FITC	Beckman Coulter (B30652)
	IgM-PacBlue	Beckman Coulter (B30656)
5	CD45-KrOrange	Beckman Coulter (B36294)
	CD1c-PC5.5	Beckman Coulter (CD)
	CD11c-PC7	Beckman Coulter (A80249)
	CD16-FITC	Beckman Coulter (IM0814U)
	CD123-APC-A700	Beckman Coulter (B24028)
	Clec9A-APC	Beckman Coulter (CD)
	HLA-DR-PacBlue	Beckman Coulter (A74781)
	LIN-PE	Beckman Coulter (B29559)
6	CD45-KrOrange	Beckman Coulter (B36294)
	CD3-APC-A750	Beckman Coulter (A94680)
	CD4-APC	Beckman Coulter (IM2468)
	CD8-APC-A700	Beckman Coulter (CD)
	CD25-PC7	Beckman Coulter (A52882)
	CXCR3-PC5.5	BioLegend (353714)
	CCR4-Brilliant Violet	BioLegend (359413)
	CCR6-FITC	BioLegend (353411)
	CCR10-PE	R&D Systems (FAB3478P)
7	CD45-KrOrange	Beckman Coulter (A96416)
	CD3-PacBlue	Beckman Coulter (A93687)
	CD4-PC7	Beckman Coulter (737660)
	CD6-FITC	Beckman Coulter (B16492)
	CD25-PE	Beckman Coulter (A07774)
	CD127-APC-A700	Beckman Coulter (A71116)
	FoxP3-APC	BioLegend (30650, Clone 259D)

Suppl. table 2: List of TaqMan gene expression assays used for Microfluidic Custom TaqMan® Array Cards.

No.	Gene Symbol	Assay ID
1	HPRT1	Hs02800695_m1
2	TLR5	Hs01019558_m1
3	PNOC	Hs00918595_m1
4	MS4A1	Hs00544818_m1
5	TCL1A	Hs00172040_m1
6	HS3ST1	Hs01099196_m1
7	SH2D1B	Hs01592483_m1
8	FCRL1	Hs00957541_m1
9	FCRL2	Hs00229156_m1
10	SLC8A1	Hs01062258_m1
11	GAPDH	Hs99999905_m1
12	CD79B	Hs00236881_m1
13	B2M	Hs00984230_m1
14	CD247	Hs00167901_m1
15	FOXP3	Hs00203958_m1
16	MAN1A1	Hs00195458_m1
17	C3orf23	Hs00603313_m1
18	TMEM176B	Hs00962650_m1
19	NAV3	Hs00372108_m1
20	CD274	Hs01125301_m1
21	CXCL10	Hs99999049_m1
22	CD200	Hs01033303_m1
23	HMMR	Hs00234864_m1
24	LAG3	Hs00158563_m1

Suppl. table 3: Means with standard error of mean of all analysed immune cell subset in proportions. The first column shows the collation between healthy controls and the three ITx groups split up according to their time period since transplantation. In the second column, the comparison of the ITx patients among themselves corresponding to their rejection status can be seen. The fitting p-values for each relation are shown. Significant values are highlighted.

**frequencies**	**Healthy Control vs. Small Bowel Transplantation**								**Acute Rejection (AR)**						
	**Mean +/- SEM**				**Statistics**				**Mean +/- SEM**			**Statistics**			
**Cell subset**	**Healthy controls**	**Tx > 10 years (ITx 1)**	**Tx 4-10 years (ITx 2)**	**Tx < 4 years (ITx 3)**	**Kuskal-Wallis-Test**	**post-hoc test after Conover**			**No AR (0)**	**Humoral/ Mixed AR (1)**	**Cellular AR (2)**	**Kuskal-Wallis-Test**	**post-hoc test after Conover**		
						**HC vs. 1**	**HC vs. 2**	**HC vs. 3**					**0 vs.1**	**0 vs. 2**	**1 vs. 2**
**Granulocytes**	61.60±1.90	63.56±3.31	61.77±3.62	62.19±5.75	0.8922	0.6208	0.6070	0.5393	64.59±2.85	65.60±4.24	51.89±4.74	*0.0627*	0.6347	*0.0507*	**0.0212**
**CD14^{+} Monocytes**	7.25±0.39	9.83±0.85	8.39±0.63	9.42±1.30	*0.0613*	**0.0137**	0.1711	*0.0646*	8.94±0.57	8.70±0.95	9.37±0.58	0.6166	0.6195	0.6326	0.3564
CD14^{+} CD16^{high}	4.50±0.43	3.54±0.50	4.99±0.84	7.53±1.61	0.2319	0.2921	0.7034	0.1614	**3.28±0.68**	**4.32±0.66**	**8.02±1.52**	**0.0257**	0.1502	**0.0044**	*0.0713*
CD14^{high} CD16^{+}	**5.11±0.64**	**6.80±1.03**	**8.80±0.86**	**8.33±1.19**	**0.0059**	0.0957	**0.0010**	**0.0041**	6.08±0.78	7.76±0.80	9.87±1.22	*0.0747*	0.2031	**0.0219**	0.2029
CD14^{high} CD16^{-}	88.96±0.98	88.76±1.46	85.01±1.60	83.02±2.50	*0.0752*	0.9703	*0.0532*	**0.0321**	**89.55±1.35**	**86.71±1.42**	**81.12±1.77**	**0.0257**	0.1502	**0.0044**	*0.0713*
**Dendritic cells**	0.71±0.06	0.83±0.17	0.82±0.09	1.21±0.34	0.3014	0.5511	0.3580	*0.0668*	**0.65±0.14**	**0.72±0.08**	**1.67±0.34**	**0.0071**	1.0000	**0.0012**	**0.0012**
pDCs	24.35±2.87	20.34±8.30	11.95±2.39	17.65±6.90	**0.0184**	*0.0557*	**0.0031**	**0.0321**	19.51±7.03	17.10±7.80	12.24±2.75	0.8687	0.6263	0.7644	0.8839
mDCs	75.65±2.87	79.66±8.30	88.05±2.39	82.35±6.90	**0.0184**	*0.0557*	**0.0031**	**0.0321**	80.49±7.03	82.90±7.80	87.76±2.75	0.8687	0.6263	0.7644	0.8839
CD16^{+} mDCs	**67.03±2.90**	**71.26±6.08**	**77.30±3.47**	**76.60±12.49**	**0.0239**	0.4218	*0.0658*	**0.0025**	66.96±11.45	71.30±5.57	86.22±1.69	*0.0650*	0.7121	**0.0467**	**0.0238**
CD1c^{+} mDCs	**30.64±2.69**	**25.18±6.35**	**20.79±3.47**	**21.72±11.66**	**0.0249**	0.1961	*0.0577*	**0.0028**	29.38±11.05	26.36±5.52	12.80±1.64	0.1651	0.4636	0.2096	*0.0636*
Clec9^{+} mDCs	1.16±0.24	1.02±0.37	0.77±0.18	1.05±0.53	0.4898	0.4990	0.2421	0.2073	1.30±0.50	1.06±0.35	0.48±0.08	0.3205	0.7775	0.1635	0.2482
**Lymphocytes**	30.55±1.76	26.24±2.92	28.82±3.32	27.82±5.46	0.5489	0.2999	0.4372	0.2429	25.89±2.70	24.79±3.79	37.85±4.96	*0.0599*	0.5607	*0.0549*	**0.0188**
CD56^{+}NK cells	10.83±1.12	12.45±2.03	13.25±1.72	15.72±2.52	0.2965	0.5923	0.2158	*0.0830*	15.80±2.47	13.89±1.31	9.55±2.41	0.1169	0.6869	**0.0476**	*0.0957*
CD56^{high}	6.69±1.01	10.81±1.93	8.35±1.01	14.11±3.79	0.1266	*0.0793*	0.3112	**0.0395**	6.53±1.21	10.17±1.27	12.63±2.57	*0.0728*	*0.0811*	**0.0279**	0.4828
CD56^{dim}	93.31±1.01	89.19±1.93	91.65±1.01	85.89±3.79	0.1266	*0.0793*	0.3112	**0.0395**	93.47±1.21	89.83±1.27	87.37±2.57	*0.0728*	*0.0811*	**0.0279**	0.4828
CD3^{+} T cells	**74.09±1.20**	**71.88±2.14**	**62.11±2.51**	**58.87±5.99**	**0.0015**	0.3738	**0.0003**	**0.0006**	58.10±4.22	67.38±2.44	67.27±7.82	0.2395	0.1378	0.1834	0.9720
CD56^{+}	10.01±1.52	9.69±3.65	8.03±2.61	14.96±5.02	0.7506	0.8433	0.3599	0.8222	7.51±3.24	12.51±3.87	5.43±1.00	0.2374	0.1265	0.8883	0.2025
TCR ab	91.08±3.76	91.85±2.18	89.44±1.85	88.19±2.75	*0.0809*	0.5586	**0.0432**	**0.0281**	93.29±2.67	87.85±1.95	89.12±2.37	0.1762	*0.0917*	0.1504	0.9002
TCR gd	5.44±1.27	7.65±3.10	9.18±1.87	11.30±2.66	0.0921	0.8050	*0.0889*	**0.0246**	6.02±2.63	11.29±2.27	8.85±2.46	0.2257	0.1056	0.2446	0.7251
CD4^{+}	1.04±0.35	0.79±0.27	0.49±0.20	1.57±1.08	0.7165	1.0000	0.2895	0.6776	1.01±0.33	0.42±0.16	1.82±1.52	0.4376	0.2227	0.5183	0.6256
CD8^{+}	29.85±2.53	31.09±3.73	27.91±6.99	25.44±5.40	0.7313	0.7571	0.4603	0.4979	27.82±7.28	28.17±4.97	26.32±6.44	0.9857	0.8947	0.9871	0.8911
CD4^{-}CD8^{-}	69.07±2.55	68.16±3.54	71.23±6.85	73.08±5.01	0.6582	0.7261	0.3865	0.4818	71.11±7.04	71.27±5.00	71.73±5.58	0.9170	0.8594	0.6986	0.8210
Vd1^{+}	**25.56±3.93**	**68.87±9.90**	**38.04±10.36**	**51.64±11.06**	**0.0162**	**0.0016**	0.3467	*0.0507*	63.00±5.95	41.52±11.42	55.77±18.97	0.5979	0.3939	1.0000	0.4355
Vd2^{+}	**61.40±4.76**	**12.64±8.39**	**47.04±13.44**	**38.32±13.12**	**0.0083**	**0.0004**	0.4540	0.1154	13.77±3.64	45.96±13.63	35.13±20.80	0.4965	0.3202	0.9866	0.3545
CD4^{+} (TCRab)	**61.95±2.44**	**48.96±7.34**	**46.20±3.14**	**35.60±7.76**	**0.0068**	0.1103	**0.0034**	**0.0016**	47.44±7.37	41.34±5.20	45.19±8.63	0.7188	0.4493	0.7869	0.6707
HLA-DR^{+}	**8.26±0.85**	**21.29±8.28**	**17.62±2.44**	**31.32±6.71**	**0.0004**	**0.0084**	**0.0004**	**<0.0001**	18.84±5.61	26.08±6.88	24.99±8.70	0.3727	0.1877	0.3945	0.7081
CD27^{-}	**8.05±1.23**	**20.61±11.67**	**20.08±4.08**	**30.06±7.17**	**0.0065**	0.3712	**0.0088**	**0.0008**	18.04±6.18	31.15±9.56	20.15±9.75	0.4440	0.2577	0.8993	0.3598
CD27^{-}PD1^{+}	**39.47±3.50**	**61.89±6.26**	**54.19±7.46**	**59.94±6.29**	**0.0116**	**0.0057**	*0.0667*	**0.0043**	61.89±7.30	57.48±7.38	52.59±7.79	0.5979	0.3939	0.4355	1.0000
CD28^{-}	1.61±0.67	12.99±10.96	4.13±1.51	14.18±5.24	*0.0719*	0.3326	0.2880	**0.0083**	6.17±2.85	13.89±9.83	8.84±7.56	0.9786	0.9297	0.9102	0.8467
CD27^{-} CD28^{-}	1.54±0.66	12.70±10.83	3.96±1.48	13.94±5.21	*0.0719*	0.3278	0.3532	**0.0082**	6.02±2.82	13.56±9.71	8.65±7.53	0.9946	0.9648	0.9239	0.9551
CD27^{+} PD1^{+}	**16.95±1.56**	**32.78±6.35**	**36.01±2.18**	**46.23±6.29**	**<0.0001**	**0.0005**	**<0.0001**	**<0.0001**	38.99±6.91	38.97±5.10	32.92±3.81	0.7973	0.7215	0.7633	0.5328
CD57^{+}	**2.94±0.85**	**12.70±8.76**	**6.29±1.70**	**17.53±5.49**	**0.0103**	0.1323	**0.0442**	**0.0007**	8.62±3.31	14.37±7.75	11.95±8.10	0.9786	0.9297	0.9102	0.8467
CCR7^{+} CD45RA^{+} (T_N)	**41.38±3.36**	**22.81±5.47**	**19.25±3.13**	**13.04±5.15**	**0.0011**	**0.0155**	**0.0028**	**<0.0001**	24.34±5.72	16.01±4.61	15.23±2.81	0.3891	0.2925	0.2344	0.8119
CCR7^{+} CD45RA^{-} (T_{CM})	41.22±3.04	47.02±20.21	50.77±8.24	39.03±14.41	0.1673	0.1801	*0.0829*	0.7034	46.53±5.01	41.78±6.29	51.71±7.05	0.2528	0.5999	0.2513	0.1126
CCR7^{-} CD45RA^{-} (T_{EM})	**15.41±1.50**	**27.11±25.44**	**28.98±12.03**	**43.22±18.63**	**0.0023**	0.1744	**0.0055**	**0.0001**	26.87±7.82	36.11±7.41	31.38±8.03	0.4130	0.2053	0.5552	0.5552
CCR7^{-} CD45RA^{+} (T_{EMRA})	1.99±1.26	2.85±2.31	1.29±0.63	4.18±1.77	0.3420	0.9409	0.9693	0.1010	2.15±1.82	3.51±2.09	1.29±0.81	0.7938	0.5937	0.5811	0.9480
CD25$^{-/+}$CCR4^{+} CCR6^{-} CXCR3^{-} CCR10^{-} (Th_2)	5.11±0.64	8.38±0.89	6.95±0.91	6.98±1.50	0.1059	**0.0206**	0.1320	0.2720	8.27±1.22	6.91±1.21	7.65±1.18	0.7365	0.4766	0.8635	0.6303
CD25$^{-/+}$CCR4^{-} CCR6^{-} CXCR3^{+}CCR10^{-} (Th_1)	8.46±0.76	14.02±4.44	8.82±1.37	12.37±3.18	0.5973	0.3672	0.9772	0.2965	8.05±1.33	12.47±4.23	14.12±4.23	0.5538	0.5860	0.3061	0.5901
CD25$^{-/+}$CCR4^{-}CCR6^{+} CXCR3^{-} CCR10^{-} (Th_{17})	0.97±0.13	1.12±0.33	1.26±0.22	0.81±0.13	0.5599	0.9768	0.3504	0.4810	1.21±0.29	1.05±0.30	0.88±0.17	0.6421	0.4731	0.4280	0.8884
CD25$^{-/+}$CCR4^{+}CCR6^{+} CXCR3^{-} CCR10^{-} (Th_{22})	**4.85±0.55**	**15.82±1.78**	**11.80±1.48**	**15.67±3.75**	**0.0001**	**<0.0001**	**0.0002**	**0.0001**	15.86±1.62	12.69±2.77	16.28±4.15	0.8356	0.5948	0.6911	0.9287

$CD25^{-/+}CCR4^{+}CCR6^{-}$ $CXCR3^{+}CCR10^{-}$ (Th_1/Th_2)	**6.88±0.80**	**11.22±1.56**	**13.64±1.46**	**8.00±0.67**	**0.0017**	**0.0055**	**<0.0001**	0.2029	10.84±1.47	10.22±1.32	13.45±2.45	0.2014	0.1453	0.1203	0.8107
$CD25^{-/+}CCR4^{-}$ $CCR6^{+}$ $CXCR3^{+}CCR10^{-}$ (pathogenic Th_{17})	4.83±0.64	5.03±1.02	10.63±2.62	7.92±2.56	*0.0602*	0.7252	**0.0074**	0.2658	5.66±1.30	10.20±3.30	5.86±0.88	0.3540	0.2012	0.9114	0.2860
$CD25^{-/+}CCR4^{+}CCR6^{+}$ $CXCR3^{+}$ $CCR10^{-}$	**4.62±0.68**	**10.37±1.40**	**11.64±0.93**	**12.39±2.05**	**0.0006**	**0.0026**	**0.0002**	**0.0002**	10.98±1.41	11.87±2.08	12.17±1.25	0.1934	0.2920	*0.0796*	0.3934
$CD25^{++}$ $FoxP3^{+}$ (T_{reg})	**5.89±0.49**	**9.97±1.35**	**8.19±0.61**	**10.74±1.02**	**0.0010**	**0.0025**	**0.0143**	**<0.0001**	8.76±0.67	9.80±1.33	8.77±1.04	0.7726	0.5053	0.8193	0.7018
$CD25^{++}CCR4^{+}$ (activated/memory Treg)	**67.24±1.90**	**85.27±3.56**	**87.13±2.23**	**90.82±1.51**	**<0.0001**	**0.0148**	**0.0011**	**0.0001**	85.07±3.91	90.90±1.83	87.04±0.62	0.2755	0.1660	0.9930	0.2010
$CD25^{++}CCR4^{+}CCR6^{-}$ $CXCR3^{+}CCR10^{-}$ (T_{reg}: Th_1)	9.01±0.92	7.17±1.02	8.27±1.30	5.93±1.04	0.3305	0.3574	0.8448	*0.0909*	6.83±1.54	8.01±1.03	7.10±1.44	0.8782	0.6902	0.9612	0.6801
$CD25^{++}CCR4^{+}CCR6^{-}$ $CXCR3^{+}CCR10^{+}$ (T_{reg}: Th_1 - 2)	2.88±0.47	1.98±0.39	2.17±0.44	1.67±0.34	0.5931	0.4740	0.5902	0.2020	1.58±0.30	2.70±0.44	1.48±0.43	0.1071	*0.0611*	0.9482	*0.0750*
$CD25^{++}CCR4^{+}CCR6^{-}$ $CXCR3^{-}CCR10^{-}$ (T_{reg}: Th_2)	11.30±0.98	9.89±1.61	12.92±1.77	13.51±2.20	0.5206	0.4607	0.4293	0.4757	11.62±1.31	11.60±1.99	10.80±2.42	0.9295	0.9295	0.7287	0.7899
$CD25^{++}CCR4^{+}CCR6^{-}$ $CXCR3^{-}CCR10^{+}$ (T_{reg}: Th_2 - 2)	4.79±0.66	3.16±0.54	2.63±0.43	4.20±0.71	*0.0776*	0.1290	**0.0173**	0.8548	2.72±0.46	3.47±0.56	2.77±0.39	0.5695	0.3241	0.7651	0.5409
$CD25^{++}CCR4^{+}CCR6^{+}$ $CXCR3^{-}CCR10^{-}$ (T_{reg}: Th_{17})	**15.18±1.01**	**21.76±2.54**	**21.08±2.24**	**24.00±1.72**	**0.0029**	**0.0148**	**0.0088**	**0.0003**	21.97±2.44	20.44±2.13	25.19±2.80	0.4016	0.7105	0.3443	0.2056
$CD25^{++}CCR4^{+}CCR6^{+}$ $CXCR3^{-}CCR10^{+}$ (T_{reg}: Th_{17} - 2)	**11.22±1.55**	**19.88±2.96**	**16.38±2.35**	**21.82±2.59**	**0.0064**	**0.0078**	*0.0518*	**0.0010**	19.93±3.48	18.92±2.52	18.87±3.38	0.9354	0.8250	0.7409	0.8972
$CD25^{++}CCR4^{+}CCR6^{+}$ $CXCR3^{+}$ $CCR10^{-}$ (T_{reg}: Th_1/Th_{17})	**8.94±0.57**	**13.58±1.52**	**15.15±1.52**	**12.64±2.49**	**0.0049**	**0.0071**	**0.0005**	0.1022	13.09±2.13	15.90±1.51	14.16±2.42	0.4356	0.2394	0.8197	0.3899
$CD25^{++}CCR4^{+}CCR6^{+}$ $CXCR3^{+}$ $CCR10^{+}$ (T_{reg}: Th_1/Th_{17} - 2)	**3.92±0.63**	**6.79±1.84**	**7.42±0.96**	**5.99±1.10**	**0.0188**	*0.0928*	**0.0023**	**0.0406**	6.51±0.87	8.87±1.51	5.51±1.40	0.2676	0.5071	0.3159	0.1175
$CD8^{+}$ (TCRab)	**32.21±2.08**	**43.60±5.62**	**44.85±2.87**	**52.83±7.91**	**0.0087**	*0.0577*	**0.0053**	**0.0022**	44.76±6.20	47.83±3.98	46.92±9.68	0.7492	0.4770	0.7439	0.7439
$CD25^{+}$	8.50±4.95	15.92±7.84	12.59±6.66	3.88±0.89	0.3240	0.1857	0.1265	0.7995	4.15±0.89	16.24±7.59	14.51±10.13	0.3480	0.1859	0.3119	0.8305
$HLA\text{-}DR^{+}$	**28.52±3.68**	**46.73±9.42**	**44.26±6.34**	**52.76±8.06**	**0.0273**	*0.0612*	**0.0297**	**0.0069**	41.85±7.47	51.02±7.86	53.24±11.03	0.6485	0.4467	0.4617	0.9671
$CD27^{-}$	25.61±3.79	38.44±12.11	30.76±8.45	47.26±12.13	0.3900	0.3836	0.9049	0.1196	36.78±12.01	38.05±10.81	38.91±16.21	0.9611	0.9648	0.8340	0.8027
$CD27^{-}PD1^{+}$	18.79±3.57	25.34±8.62	33.78±5.06	28.96±6.58	*0.0536*	0.4123	**0.0074**	0.1014	34.91±8.64	28.21±4.48	25.49±9.56	0.6996	0.8927	0.4492	0.5245
$CD28^{-}$	35.10±3.83	46.96±11.67	34.96±8.64	50.39±11.46	0.5132	0.3836	0.8438	0.1833	39.41±10.73	43.33±10.72	45.49±16.46	0.9146	0.7235	0.7528	0.9935
$CD27^{-}$ $CD28^{-}$	22.94±3.68	35.53±12.24	25.88±8.18	40.68±12.11	0.5676	0.4043	0.9831	0.2390	29.61±11.26	33.32±11.10	36.06±16.14	0.9304	0.7238	0.8717	0.8717
$CD27^{+}$ $PD1^{+}$	**27.06±2.09**	**32.54±4.76**	**38.54±5.21**	**37.45±3.01**	**0.0404**	0.2044	**0.0200**	**0.0136**	34.45±4.29	40.25±5.13	35.17±6.24	0.8394	0.6257	0.9935	0.6501
$CD57^{+}$	32.17±3.67	35.05±6.87	32.74±7.47	47.90±8.49	0.4176	0.9853	0.8786	0.1369	36.08±8.73	38.69±6.76	37.50±13.04	0.9146	0.7235	0.9935	0.7528
$CCR7^{+}$ $CD45RA^{+}$ (T_N)	30.58±3.30	25.48±6.63	29.36±5.35	13.42±4.73	*0.0983*	0.5145	0.8316	**0.0148**	27.20±8.40	21.46±4.25	22.58±7.13	0.7492	0.4770	0.7439	0.7439
$CCR7^{+}$ $CD45RA^{-}$ (T_{CM})	13.11±1.83	12.33±3.25	15.59±4.05	5.95±2.01	0.1656	0.9378	0.8273	**0.0417**	8.39±1.94	14.90±5.07	13.37±3.97	0.6166	0.6195	0.3564	0.6326
$CCR7^{-}$ $CD45RA^{-}$ (T_{EM})	31.22±3.37	22.71±4.53	27.82±2.89	42.71±4.78	0.0525	0.1190	0.4024	*0.0826*	34.85±4.70	27.98±4.36	30.81±9.16	0.6421	0.4731	0.4280	0.8884
$CCR7^{-}$ $CD45RA^{+}$ (T_{EMRA})	25.09±3.68	38.82±11.24	27.51±7.43	37.01±4.70	0.2942	0.2436	0.6696	*0.0832*	28.78±6.86	36.00±11.09	33.09±8.00	0.8687	0.7228	0.6392	0.8839
B cells	9.71±0.67	9.25±1.22	16.97±3.16	13.91±5.62	*0.0967*	0.8491	**0.0210**	0.9537	18.99±4.48	10.82±1.79	15.42±5.46	0.3097	0.1440	0.3983	0.6002
Transitional	2.79±0.37	7.84±3.58	2.55±0.44	3.66±1.38	0.3724	0.1191	0.8902	0.9120	3.12±1.31	6.83±3.35	3.17±1.13	0.2279	*0.0969*	0.5344	0.3442
Naive	54.76±3.00	54.34±4.88	69.27±4.89	62.90±6.83	0.1315	0.9755	**0.0307**	0.2112	67.03±7.69	64.61±5.11	63.45±2.89	0.8501	0.7559	0.5992	0.8077
Marginal Zone	13.51±1.78	10.32±0.89	7.76±1.45	7.89±1.78	*0.0769*	0.6670	**0.0277**	**0.0442**	6.76±1.15	9.38±1.46	7.55±1.75	0.4586	0.2408	0.6992	0.4828
Class-non-switched memory	**4.08±0.56**	**2.05±0.65**	**1.21±0.37**	**1.23±0.25**	**0.0006**	**0.0113**	**0.0001**	**0.0002**	1.74±0.73	1.06±0.21	1.46±0.30	0.6398	0.8565	0.4862	0.3916
Class-switched memory	**15.63±1.79**	**11.96±3.25**	**8.21±1.56**	**8.08±1.84**	**0.0388**	0.2460	**0.0096**	**0.0268**	10.48±3.75	7.76±1.02	8.56±0.90	0.8501	0.5951	0.8077	0.8077
Plasmablasts	1.36±0.36	1.86±0.57	1.36±0.25	1.88±0.61	0.8719	0.5640	0.5730	0.5420	1.46±0.53	1.57±0.36	1.54±0.62	0.9146	0.7235	0.9935	0.7528
$CD21^{low}CD38^{low}$	**8.78±1.20**	**15.26±2.53**	**9.09±1.69**	**16.87±4.00**	**0.0431**	**0.0161**	0.7309	**0.0330**	8.63±1.27	12.37±2.43	15.37±3.77	0.3226	0.3764	0.1544	0.5158

Suppl. table 4: Means with standard error of mean of all analysed immune cells subsets in absolute cell numbers (/nl). The first column shows the collation between healthy controls and the three ITx groups split up according to their time period since transplantation. In the second column, the comparison of the ITx patients among themselves corresponding to their rejection status can be seen. The fitting p-values for each relation are shown. Significant values are highlighted.

**Absolute cell number**	**Healthy Control vs. Small Bowel Transplantation**								**Acute Rejection (AR)**						
	**Mean +/- SEM**				**Statistics**				**Mean +/- SEM**			**Statistics**			
**Cell subset**	**Healthy controls**	**Tx > 10 years (ITx 1)**	**Tx 4-10 years (ITx 2)**	**Tx < 4 years (ITx 3)**	**Kuskal-Wallis-Test**	**post-hoc test after Conover**			**No AR (0)**	**Humoral/ Mixed AR (1)**	**Cellular AR (2)**	**Kuskal-Wallis-Test**	**post-hoc test after Conover**		
						**HC vs. 1**	**HC vs. 2**	**HC vs. 3**					**0 vs.1**	**0 vs. 2**	**1 vs. 2**
**CD45+ Leucocytes**	6.546±0.562	6.872±0.576	6.971±0.944	8.19±1.304	0.5129	0.2945	0.5818	0.1990	8.540±0.700	7.127±1.073	7.386±1.354	0.3140	0.1832	0.2411	0.9587
**Granulocytes**	4.030±0.346	4.392±0.472	4.097±0.328	4.872±0.760	0.6557	0.4401	0.6528	0.2587	5.461±0.602	4.420±0.364	3.685±0.385	*0.0919*	*0.2889*	**0.0287**	0.1800
**CD14$^+$ Monocytes**	0.481±0.060	0.662±0.078	0.603±0.093	0.708±0.107	*0.0738*	**0.0476**	0.1467	**0.0256**	0.771±0.066	0.629±0.112	0.663±0.089	0.4060	0.2049	0.6662	0.4542
CD14$^+$ CD16^{high}	0.020±0.002	0.023±0.003	0.030±0.007	0.046±0.013	0.1598	0.4180	0.2430	**0.0282**	0.023±0.005	0.029±0.008	0.055±0.015	*0.0679*	0.4649	**0.0207**	*0.0778*
CD14^{high} CD16$^+$	**0.025±0.004**	**0.045±0.006**	**0.051±0.008**	**0.055±0.011**	**0.0021**	**0.0077**	**0.0009**	**0.0008**	0.045±0.006	0.048±0.009	0.068±0.013	0.2483	0.9236	0.1371	0.1593
CD14^{high} CD16$^-$	0.430±0.056	0.576±0.067	0.520±0.081	0.599±0.099	0.1067	**0.0433**	0.2506	*0.0509*	0.677±0.062	0.552±0.097	0.541±0.071	0.4440	0.2577	0.3598	0.8993
**Dendritic cells**	0.047±0.008	0.054±0.009	0.055±0.007	0.096±0.034	0.5226	0.4685	0.2584	0.2331	0.050±0.010	0.050±0.008	0.132±0.038	0.1026	0.8784	**0.0483**	*0.0632*
pDCs	0.011±0.001	0.008±0.002	0.006±0.001	0.012±0.006	0.1452	0.2114	**0.0266**	0.3266	0.0058±0.0005	0.0064±0.0019	0.0164±0.0071	*0.7479*	0.3803	0.1075	**0.0223**
mDCs	0.036±0.007	0.045±0.010	0.049±0.007	0.084±0.030	0.1974	0.2124	*0.0557*	0.1772	**0.044±0.011**	**0.043±0.009**	**0.114±0.032**	**0.0416**	0.7019	**0.0132**	**0.0273**
CD16$^+$ mDCs	0.025±0.006	0.035±0.010	0.038±0.005	0.066±0.025	*0.0606*	0.2044	**0.0216**	**0.0298**	**0.030±0.008**	**0.032±0.007**	**0.097±0.025**	**0.0272**	0.9546	**0.0108**	**0.0097**
CD1c$^+$ mDCs	0.010±0.002	0.010±0.003	0.010±0.002	0.011±0.005	0.6432	0.6477	0.5719	0.2197	0.008±0.002	0.011±0.003	0.016±0.006	0.5194	0.6160	0.2790	0.5226
Clec9$^+$ mDCs	0.0004±0.0001	0.0003±0.0001	0.0004±0.0001	0.0005±0.0002	0.9882	0.9986	0.9883	0.7484	0.0004±0.0001	0.0004±0.0001	0.0006±0.0002	0.5788	0.8202	0.3384	0.4490
**Lymphocytes**	1.998±0.212	1.783±0.205	2.110±0.540	2.369±0.764	0.9710	0.8232	0.6547	0.9423	2.057±0.116	1.953±0.634	2.968±0.940	0.2053	0.1340	0.8942	0.1346
CD56$^+$NK cells	0.212±0.026	0.220±0.050	0.247±0.054	0.303±0.060	0.5774	0.8931	0.7287	0.2072	0.323±0.056	0.245±0.064	0.237±0.052	0.3384	0.1713	0.3393	0.7512
CD56^{high}	**0.012±0.002**	**0.020±0.003**	**0.020±0.003**	**0.034±0.006**	**0.0008**	**0.0083**	**0.0048**	**<0,0001**	0.020±0.004	0.023±0.003	0.030±0.009	0.5820	0.6504	0.3266	0.5632
CD56^{dim}	0.201±0.026	0.201±0.049	0.228±0.052	0.271±0.059	0,7897	0.8339	0.8430	0.3872	0.303±0.055	0.224±0.061	0.207±0.046	0.2327	0.1158	0.2229	0.8057
CD3$^+$ T cells	1.474±0.154	1.277±0.154	1.356±0.365	1.541±0.685	0.4050	0.6352	0.2057	0.1563	1.229±0.116	1.315±0.423	2.142±0.1	0.2501	0..3001	0.4981	0.1142
CD56$^+$	0.133±0.019	0.129±0.053	0.089±0.024	0.141±0.043	0.4741	0.4343	0.1564	0.9339	0.096±0.042	0.146±0.050	0.109±0.040	0.7726	0.5053	0.7018	0.8193
TCR ab	1.368±0.163	1.176±0.161	1.200±0.305	1.404±0.649	0.3745	0.6264	0.2281	0.1233	1.145±0.109	1.125±0.351	1.980±0.831	0.1955	0.1571	0.7292	0.1070
TCR gd	0.066±0.011	0.091±0.036	0.135±0.058	0.145±0.0.055	0.5295	0.9636	0.3104	0.2527	0.075±0.035	0.165±0.066	0.169±0.064	0.1217	*0.0923*	*0.0663*	0.7410
CD4$^+$	0.0006±0.0002	0.0002±0.0001	0.0003±0.0001	0.0021±0.0017	0.8799	0.4926	0.8612	0.8652	0.0003±0.0001	0.0004±0.0001	0.0028±0.0023	0.7465	0.8578	0.4851	0.5909
CD8$^+$	0.021±0.005	0.031±0.013	0.051±0.024	0.039±0.020	0.8886	0.7216	0.9929	0.5846	0.023±0.012	0.056±0.026	0.053±0.028	0.3546	0.2337	0.2381	0.9250
CD4$^-$CD8$^-$	0.044±0.007	0.059±0.023	0.086±0.037	0.105±0.038	0.4142	0.9912	0.2219	0.2016	0.052±0.029	0.110±0.042	0.113±0.039	0.1035	*0.0735*	*0.0580*	0.7733
Vd1$^+$	0.014±0.002	0.077±0.034	0.062±0.028	0.092±0.048	0.1271	*0.0932*	0.5284	**0.0367**	0.055±0.029	0.087±0.037	0.112±0.060	0.7299	0.7880	0.4599	0.6186
Vd2$^+$	**0.041±0.008**	**0.004±0.002**	**0.039±0.011**	**0.036±0.016**	**0.0059**	**0.0003**	0.9382	0.4923	0.009±0.004	0.039±0.012	0.036±0.024	0.1731	*0.0694*	0.5384	0.2678
CD4$^+$ (TCRab)	**0.937±0.115**	**0.612±0.109**	**0.565±0.119**	**0.438±0.138**	**0.0299**	0.1378	**0.0375**	**0.0052**	0.581±0.109	0.487±0.142	0.705±0.115	0.1652	0.4636	0.2096	*0.0636*
HLA-DR$^+$	0.075±0.011	0.089±0.012	0.099±0.030	0.156±0.078	0.6946	0.2755	0.5392	0.5507	0.079±0.009	0.111±0.034	0.207±0.102	0.4586	0.7472	0.2453	0.3780
CD27$^-$	0.066±0.009	0.070±0.023	0.110±0.038	0.133±0.070	0.7170	0.9512	0.2783	0.7393	0.069±0.017	0.126±0.044	0.164±0.096	0.6404	0.3958	0.5329	0.8754
CD27$^-$PD1$^+$	0.026±0.004	0.049±0.021	0.073±0.036	0.085±0.050	0.4880	0.4541	0.2089	0.2447	0.045±0.014	0.090±0.042	0.101±0.069	0.8687	0.6263	0.7644	0.8839
CD28$^-$	0.012±0.005	0.029±0.022	0.023±0.011	0.065±0.048	0.3762	0.4959	0.5436	*0.0891*	0.020±0.008	0.040±0.021	0.075±0.068	0.9857	0.8947	0.9871	0.8911
CD27$^-$ CD28$^-$	0.011±0.005	0.028±0.021	0.023±0.011	0.064±0.047	0.3553	0.4251	0.5437	*0.0844*	0.019±0.008	0.040±0.021	0.740±0.068	1.0000	1.0000	1.0000	1.0000
CD27$^+$ PD1$^+$	0.127±0.013	0.131±0.026	0.154±0.029	0.112±0.026	0.6896	0.6294	0.4266	0.6383	0.153±0.022	0.118±0.036	0.161±0.017	0.1290	0.1338	0.5539	*0.0575*
CD57$^+$	0.023±0.006	0.037±0.016	0.039±0.019	0.084±0.055	0.4627	0.4620	0.5887	0.1281	0.030±0.009	0.053±0.023	0.102±0.078	0.8687	0.7228	0.6392	0.8839
CCR7$^+$ CD45RA$^+$ (T_N)	**0.372±0.048**	**0.161±0.052**	**0.122±0.037**	**0.052±0.019**	**0.0001**	**0.0030**	**0.0001**	**<0.0001**	0.159±0.050	0.097±0.044	0.095±0.012	0.3857	0.1886	0.4886	0.5935
CCR7$^+$ CD45RA$^-$ (T_{CM})	0.396±0.069	0.302±0.068	0.264±0.045	0.176±0.068	*0.0951*	0.5806	0.2726	**0.0129**	0.284±0.068	0.193±0.053	0.336±0.059	0.1743	0.2487	0.4145	*0.0715*
CCR7$^-$ CD45RA$^-$ (T_{EM})	0.134±0.020	0.111±0.018	0.156±0.043	0.186±0.073	0.9393	0.7981	0.6754	0.8936	0.109±0.019	0.168±0.048	0.239±0.092	0.4149	0.3598	0.2300	0.7036
CCR7$^-$ CD45RA$^+$ (T_{EMRA})	0.014±0.008	0.007±0.005	0.008±0.005	0.014±0.007	0.5113	0.5699	0.4194	0.4235	0.007±0.006	0.013±0.006	0.010±0.007	0.8532	0.9291	0.6737	0.6161
CD25$^{-/+}$ CCR4$^+$ CCR6$^-$ CXCR3$^-$CCR10$^-$ (Th_2)	0.0462±0.0077	0.0520±0.0116	0.0415±0.0100	0.0342±0.0140	0.6283	0.6695	0.8215	0.2997	0.0498±0.0117	0.0360±0.0119	0.0528±0.0150	0.4929	0.3419	0.9066	0.3259
CD25$^{-/+}$ CCR4$^-$ CCR6$^-$ CXCR3$^+$ CCR10$^-$ (Th_1)	0.0746±0.0094	0.0648±0.0116	0.0597±0.0223	0.0647±0.0371	0.1648	0.7292	0.1390	**0.0439**	0.0464±0.0090	0.0584±0.0257	0.1104±0.0458	0.3608	0.7436	0.2939	0.2939
CD25$^{-/+}$CCR4$^-$CCR6$^+$ CXCR3$^-$ CCR10$^-$ (Th_{17})	0.0081±0.0012	0.0083±0.0036	0.0063±0.0010	0.0034±0.0012	0.1421	0.5043	0.5510	**0.0212**	0.0087±0.0033	0.0044±0.0011	0.0058±0.0012	0.4929	0.3419	0.9066	0.3259
CD25$^{-/+}$CCR4$^+$CCR6$^+$ CXCR3$^-$ CCR10$^-$ (Th_{22})	0.0418±0.0058	0.0556±0.201	0.0542±0.0303	0.0486±0.0259	0.2716	0.7102	0.2110	0.3013	0.0651±0.0158	0.0400±0.0145	0.0722±0.0324	0.3998	0.2370	0.9458	0.3082
CD25$^{-/+}$CCR4$^+$CCR6$^-$ CXCR3$^+$CCR10$^-$ (Th_1/Th_2)	0.0621±0.0100	0.0425±0.0105	0.0527±0.0166	0.0516±0.0193	0.5393	0.2631	0.2920	0.3590	0.0486±0.0080	0.0434±0.0188	0.0752±0.0234	0.1877	0.1694	0.6502	0.0948

CD25$^{-/+}$ CCR4^{-} CCR6^{+} CXCR3^{+}CCR10^{-} (pathogenic Th$_{17}$)	0.0404±0.0068	0.0300±0.0060	0.0466±0.0046	0.0244±0.0066	*0.0709*	0.5966	*0.0983*	0.1327	0.0315±0.0069	0.0384±0.0082	0.0395±0.0056	0.9422	0.7572	0.8275	0.9485
CD25$^{-/+}$ CCR4^{+} CCR6^{+} CXCR3^{+}CCR10^{-}	0.0399±0.0066	0.0307±0.0067	0.0474±0.0081	0.0338±0.0103	0.4756	0.6166	0.2911	0.5837	0.0411±0.0063	0.0355±0.0108	0.0499±0.0085	0.4228	0.6116	0.4214	0.2127
CD25^{++} FoxP3^{+} (T$_{reg}$)	0.055±0.008	0.053±0.008	0.043±0.005	0.044±0.012	0.5736	0.7811	0.5224	0.2555	0.0489±0.0067	0.0408±0.0069	0.0616±0.0132	0.4298	0.4356	0.6017	0.2249
CD25^{++}CCR4^{+} (activated/memory Treg)	**0.0594±0.0095**	**0.0219±0.0039**	**0.0202±0.0026**	**0.0196±0.0039**	**0.0006**	**0.0021**	**0.0002**	**0.0002**	0.0197±0.0023	0.0206±0.0042	0.0251±0.0037	0.4240	0.8530	0.3061	0.2368
CD25^{++}CCR4^{+}CCR6^{-} CXCR3^{+}CCR10^{-} (T$_{reg}$: Th$_{1}$)	**0.0075±0.0012**	**0.0019±0.0004**	**0.0019±0.0004**	**0.0014±0.0004**	**<0.0001**	**0.0001**	**0.0001**	**<0.0001**	0.0017±0.0005	0.0019±0.0005	0.0020±0.0005	0.8966	0.7904	0.6687	0.8523
CD25^{++}CCR4^{+}CCR6^{-} CXCR3^{+}CCR10^{+} (T$_{reg}$: Th$_{1}$ - 2)	**0.0024±0.0005**	**0.0005±0.0001**	**0.0005±0.0001**	**0.0004±0.0001**	**<0.0001**	**0.0001**	**<0.0001**	**<0.0001**	0.0004±0.0001	0.0006±0.0001	0.0004±0.0001	0.3998	0.2370	0.9458	0.3082
CD25^{++}CCR4^{+}CCR6^{-} CXCR3^{-}CCR10^{-} (T$_{reg}$: Th$_{2}$)	**0.0096±0.0014**	**0.0028±0.0005**	**0.0030±0.0005**	**0.0028±0.0007**	**0.0025**	**0.0079**	**0.0019**	**0.0007**	0.0030±0.0005	0.0027±0.0006	0.0032±0.0009	0.9088	0.6907	0.7959	0.9162
CD25^{++}CCR4^{+}CCR6^{-} CXCR3^{-}CCR10^{+} (T$_{reg}$: Th$_{2}$ - 2)	**0.0045±0.0011**	**0.0007±0.0001**	**0.0006±0.0001**	**0.0008±0.0001**	**<0.0001**	**<0.0001**	**<0.0001**	**<0.0001**	0.0006±0.0001	0.0007±0.0001	0.0008±0.0002	0.6462	0.5893	0.3875	0.7049
CD25^{++}CCR4^{+}CCR6^{+} CXCR3^{-}CCR10^{-} (T$_{reg}$: Th$_{17}$)	**0.0139±0.0027**	**0.0057±0.0010**	**0.0051±0.0011**	**0.0053±0.0013**	**0.0210**	*0.0837*	**0.0057**	**0.0151**	0.0049±0.0005	0.0052±0.0014	0.0072±0.0015	0.3205	0.7775	0.2482	0.1635
CD25^{++}CCR4^{+}CCR6^{+} CXCR3^{-}CCR10^{+} (T$_{reg}$: Th$_{17}$ - 2)	0.0106±0.0027	0.0049±0.0010	0.0037±0.0007	0.0046±0.0009	0.1223	0.1782	**0.0278**	0.1458	0.0042±0.0009	0.0040±0.0006	0.0058±0.0014	0.5947	0.9638	0.4030	0.3809
CD25^{++}CCR4^{+}CCR6^{+} CXCR3^{+} CCR10^{-} (T$_{reg}$: Th1/Th$_{17}$)	**0.0076±0.0012**	**0.0033±0.0006**	**0.0037±0.0008**	**0.0031±0.0009**	**0.0085**	**0.0125**	**0.0198**	**0.0019**	0.0033±0.0008	0.0037±0.0009	0.0039±0.0007	0.7072	0.6223	0.4447	0.7493
CD25^{++}CCR4^{+}CCR6^{+} CXCR3^{+}CCR10^{+} (T$_{reg}$: Th1/Th$_{17}$ - 2)	0.0032±0.0007	0.017±0.0004	0.0017±0.0002	0.0013±0.0004	0.1090	0.1481	0.1623	**0.0239**	0.0016±0.0003	0.0018±0.0002	0.0016±0.0004	0.7286	0.5919	0.8250	0.4795
CD8^{+} (TCRab)	0.458±0.049	0.573±0.127	0.651±0.213	0.962±0.567	0,8258	0.5642	0.7049	0.6795	0.533±0.076	0.680±0.248	1.293±0.774	0.8966	0.7904	0.8523	0.6687
CD25^{+}	0.035±0.018	0.066±0.025	0.046±0.015	0.020±0.004	*0.0593*	**0.0297**	**0.0240**	0.5434	0.024±0.005	0.056±0.016	0.067±0.032	0.2327	0.1158	0.2229	0.8057
HLA-DR^{+}	0.138±0.024	0.316±0.109	0.334±0.150	0.756±0.543	0.2428	0.1908	0.1143	0.1310	0.246±0.072	0.402±0.175	1.021±0.750	0.8734	0.8244	0.6279	0.7768
CD27^{-}	0.111±0.017	0.273±0.118	0.260±0.131	0.742±0.550	0.8795	0.5417	0.9846	0.5888	0.242±0.096	0.325±0.157	0.954±0.770	0.9439	0.7910	0.9742	0.7839
CD27^{-}PD1^{+}	0.018±0.005	0.041±0.020	0.063±0.028	0.155±0.109	0.1609	0.3198	*0.0717*	*0.0639*	0.059±0.022	0.071±0.032	0.175±0.157	0.7072	0.7197	0.6469	0.4352
CD28^{-}	0.161±0.024	0.321±0.125	0.275±0.127	0.754±0.559	0.9446	0.6510	0.8803	0.7844	0.248±0.093	0.346±0.152	1.006±0.778	0.9146	0.7235	0.7528	0.9935
CD27^{-} CD28^{-}	0.099±0.017	0.256±0.114	0.225±0.118	0.692±0.536	0.8758	0.6130	0.8000	0.6442	0.200±0.090	0.292±0.144	0.914±0.748	0.8687	0.6263	0.8839	0.7644
CD27^{+} PD1^{+}	0.092±0.014	0.075±0.010	0.121±0.025	0.092±0.018	0.3943	0.8293	0.1259	0.6203	0.093±0.006	0.110±0.031	0.112±0.021	0.7072	0.7197	0.6469	0.4352
CD57^{+}	0.151±0.025	0.225±0.081	0.251±0.114	0.695±0.518	0.8240	0.7060	0.7983	0.4810	0.217±0.079	0.296±0.127	0.886±0727	0.9955	0.9297	0.9679	0.9679
CCR7^{+} CD45RA^{+} (T$_{N}$)	**0.122±0.011**	**0.114±0.023**	**0.161±0.054**	**0.049±0.012**	**0.0122**	0.6228	0.6066	**0.0008**	0.115±0.035	0.127±0.063	0.100±0.082	0.7286	0.5919	0.8250	0.4795
CCR7^{+} CD45RA^{-} (T$_{CM}$)	0.061±0.013	0.055±0.014	0.066±0.013	0.029±0.008	0.1418	0.8363	0.3276	*0.0856*	0.039±0.007	0.058±0.018	0.071±0.012	*0.0800*	0.3067	**0.0238**	0.1449
CCR7^{-} CD45RA^{-} (T$_{EM}$)	0.148±0.027	0.113±0.020	0.142±0.028	0.550±0.381	0.9308	0.7383	0.8098	0.7063	0.190±0.049	0.137±0.028	0.651±0.542	0.8974	0.9646	0.6864	0.7161
CCR7^{-} CD45RA^{+} (T$_{EMRA}$)	0.113±0.018	0.278±0.117	0.250±0.134	0.326±0.172	0.6540	0.4182	0.8523	0.2829	0.179±0.058	0.330±0.163	0.439±0.247	0.8532	0.9291	0.6737	0.6161
B cells	0.205±0.031	0.161±0.029	0.404±0.140	0.346±0.152	0.6175	0.6785	0.3014	0.6938	0.390±0.092	0.268±0.152	0.400±0.189	*0.0788*	**0.0272**	0.5834	0.1164
Transitional	0.0059±0.0011	0.0083±0.0025	0.0098±0.0040	0.0077±0.0026	0.7563	0.4028	0.4090	0.5974	0.0094±0.0026	0.0107±0.0047	0.0079±0.0016	0.7880	0.8234	0.6606	0.5221
Naive	0.1137±0.0188	0.0851±0.0141	0.3109±0.1232	0.2549±0.1121	0.2718	0.6079	0.1213	0.3703	0.2930±0.0986	0.1983±0.1234	0.2671±0.1370	0.2257	0.1056	0.7251	0.2446
Marginal Zone	0.0250±0.0041	0.0172±0.0033	0.0210±0.0037	0.0173±0.0065	0.5601	0.4021	0.7846	0.2033	0.0225±0.0029	0.0163±0.0044	0.0235±0.0069	0.2327	0.1158	0.8057	0.2229
Class-non-switched memory	**0.0072±0.0011**	**0.0038±0.0016**	**0.0031±0.0013**	**0.0041±0.0026**	**0.0152**	**0.0484**	**0.0074**	**0.0067**	0.0048±0.0019	0.0018±0.0004	0.0060±0.0033	--0.3962	0.3824	0.6294	0.2081
Class-switched memory	0.0351±0.0085	0.0255±0.0110	0.0261±0.0094	0.0258±0.0150	0.4167	0.3169	0.3442	0.1398	0.0322±0.0114	0.0188±0.0093	0.0367±0.0188	0.3436	0.3543	0.5850	0.1725
Plasmablasts	0.0028±0.0012	0.0033±0.0012	0.0045±0.0019	0.0079±0.0062	0.7002	0.6156	0.2683	0.8754	0.0041±0.0011	0.0040±0.0023	0.0109±0.0086	0.6407	0.3958	0.8754	0.5329
CD21^{low}CD38^{low}	0.0159±0.0024	0.0229±0.0038	0.0283±0.0080	0.0437±0.0228	0.3293	0.1836	0.1289	0.3093	0.0290±0.0034	0.0230±0.090	0.0600±0.0295	0.1495	*0.0967*	0.8776	*0.0963*

Suppl. Table 5: PCR statistics

HC versus Itx groups (0=HC, 1=ITx1, 2=ITx2, 3=ITx3)

	Group 1	Group 2	nr_TOAG	nr_CD79B	nr_CD200	nr_CD3	nr_CD274	nr_CXCL10	nr_FCRL1	nr_FCRL2	nr_HMMR	nr_HS3ST1	nr_LAG3	nr_aMann	nr_MS4A1	nr_NAV3	nr_PNOC	nr_SH2D1B	nr_SLC8A1	nr_TCL1A	nr_TLR5	nr_TORID
Kruskal-Wallis-Test		p.value	0.006912896	0.007259904	0.051327885	0.001422932	0.00015262	0.989880728	0.014553899	0.126241109	0.22980212	0.004185676	0.35036466	0.000833333	0.023212252	0.095524154	0.949534067	0.004363819	2.75965E-06	0.029486177	1.6223E-05	0.068805493
post-hoc test after Conover	1	0	0.038302329	0.29457875	0.236170061	0.004716779	0.003256784	0.801573066	0.295132774	0.080542317	0.528636766	0.025212668	0.397839554	0.015434994	0.617412091	0.716506787	0.632076948	0.039453217	6.104E-06	0.823222311	2.00627E-05	0.738351365
post-hoc test after Conover	2	0	0.000542289	0.000283086	0.049174814	0.000404915	4.49292E-06	0.793576828	0.000858649	0.416741837	0.092760441	0.000197197	0.149348907	9.61328E-05	0.004415493	0.13401109	0.685266346	0.000462481	1.36221E-09	0.00403883	2.37402E-07	0.154920079
post-hoc test after Conover	3	0	0.032950882	0.25675768	0.416553	0.00070443	4.04E-05	0.947542174	0.464118292	0.279547476	0.978241171	0.084052046	0.159812101	0.000331562	0.934815634	0.037686869	0.752515998	0.006840955	2.3402E-10	0.592857818	1.59462E-07	0.071274892
post-hoc test after Conover	2	1	0.26361353	0.029218675	0.011271059	0.652587555	0.132389684	0.989441679	0.05737828	0.031992869	0.061611036	0.218584317	0.678376173	0.21435158	0.007109999	0.132695498	0.920689897	0.241078606	0.076603742	0.012238351	0.454647651	0.155946396
post-hoc test after Conover	3	1	0.964340865	0.982203501	0.717071904	0.672542523	0.278240774	0.871731162	0.758541766	0.519817094	0.575414266	0.57795905	0.670462134	0.303231026	0.622520598	0.045152523	0.877117827	0.622826321	0.014011916	0.534275355	0.288659629	0.232543929
post-hoc test after Conover	3	2	0.22546094	0.024628745	0.021830773	0.98762555	0.674016691	0.873071218	0.022758478	0.111367968	0.16790333	0.064348771	0.980247747	0.845269991	0.019357983	0.498538452	0.950291934	0.481661816	0.385532734	0.044511927	0.714065905	0.007940502

noAR versus rejection groups (0=noAR, 1=AR1, 2=AR2)

	Var1	Var2	nr_TOAG	nr_CD79B	nr_CD200	nr_CD3	nr_CD274	nr_CXCL10	nr_FCRL1	nr_FCRL2	nr_HMMR	nr_HS3ST1	nr_LAG3	nr_aMann	nr_MS4A1	nr_NAV3	nr_PNOC	nr_SH2D1B	nr_SLC8A1	nr_TCL1A	nr_TLR5	nr_TORID
Kruskal-Wallis-Test		p.value	0.648506854	0.597927533	0.878161456	0.718761588	0.788048621	0.540945854	0.662702855	0.458612858	0.985667611	0.819962853	0.908791942	0.310577035	0.942189486	0.429767344	0.111639405	0.513365651	0.224847963	0.809670569	0.031030585	0.668709185
post-hoc test after Conover	1	0	0.446651557	0.393901197	0.658012762	0.449311222	0.82336772	0.322649586	0.39668316	0.240821148	0.894731307	0.689030068	0.69072935	0.213320415	0.757217384	0.926215815	0.041707476	0.280561308	0.472744217	0.964419343	0.575679963	0.752593593
post-hoc test after Conover	2	0	0.967099649	0.435523157	0.961204829	0.670702421	0.522049975	0.442411546	0.754589454	0.699154483	0.987146704	0.838873502	0.916238842	0.198666028	0.948494094	0.253263042	0.164464894	0.511679565	0.283440498	0.576049614	0.007870493	0.401676341
post-hoc test after Conover	2	1	0.461723293	1	0.721844461	0.78693293	0.660569223	0.887314188	0.639800414	0.482786771	0.891114818	0.570861842	0.795901718	0.876621104	0.827532857	0.287916521	0.580577188	0.73213016	0.093865095	0.603756275	0.023011525	0.577406795

Suppl. Table 6: Information on parameters contributing separation upon cluster analysis

HC versus ITx patients

freq_BM05_IgM+ CD27−
freq_BM05_IgD− IgM+ CD27− CD38dim
nr_HS3ST1
nr_MS4A1
nr_CD79B
nr_TCL1A
nr_FCRL1
wbc_BM02_CD3+ Vd2+ CD4+ T cells
wbc_BM05_IgD− IgM+ CD27+ CD38high
wbc_BM05_IgM+ CD27+ CD38high
wbc_BM05_IgD− IgM− CD27− CD38high
wbc_BM05_CD21low CD38high
freq_BM07_CD4+ CD25++ CCR4+ CCR6
freq_BM07_CD4+ CD25++ CCR4+ CCR6+ CXCR3− CCR10+
freq_BM07_CD4+ CD25++ CCR4+ CCR6+ CXCR3+ CCR10−
freq_BM07_CD4+ CD25++ CCR4+ CCR6+ CXCR3+ CCR10+
freq_BM02_CD3+ Vd1+
freq_BM03_CD4+ CD27− PD1+
freq_BM02_CD8+ HLA−DR+
freq_BM02_TCR ab CD8+ T cells
freq_BM01_CD8+ T cells
freq_BM07_CD4+CD25−/+CCR4+CCR6−CXCR3+CCR10− (Th1/Th2)
freq_BM07_CD4+ CD25++ CCR4+ CCR6+ CXCR3− CCR10−
freq_BM07_CD4+CD25−/+CCR4+CCR6+CXCR3+CCR10−
freq_BM07_CD4+CD25−/+CCR4+CCR6+CXCR3−CCR10− (Th22)
freq_BM03_CD4+ CD27+ CD28−
freq_BM03_CD4+ CD27+ PD1+
freq_BM03_CD4+ CCR7− CD45RA−
freq_BM02_CD4+ HLA−DR+
freq_BM03_CD4+ CD27−
freq_BM03_CD4+ CD28−
freq_BM03_CD4+ CD27− CD28−
freq_BM03_CD4+ CD57+
freq_BM05_CD21low CD38high
freq_BM05_CD21+ CD38high
freq_BM03_CD8 CCR7− CD45RA−
nr_TORID
IL−4
freq_BM03_CD4+ CD27− CD28+
freq_BM03_CD8+ CD27− CD28+
freq_BM02_TCR ab CD4− CD8− T cells
freq_BM06_mDC1
freq_BM06_pDC in pDCs+mDCs
freq_BM05_IgD− IgM+ CD27+ CD38high
IL−8
freq_BM07_CD4+ CD25−/+ CCR4− CCR6+ CXCR3+CCR10− (pathogenic Th17)
FoxP3dem_A
freq_BM08_CD4+ CD25++ FoxP3+
freq_BM08_CD25++ CD127− AND CD6−
freq_BM08_CD25++ CD127− OR CD6−
freq_BM08_CD4+ CD25++ CD127−
nr_TLR5
nr_SLC8A1
nr_aMann
nr_CD274
freq_BM03_CD8+ CD27− PD1+
nr_SH2D1B
nr_TOAG
nr_CD3
freq_BM01_CD14high CD16+ Monocytes
wbc_BM01_CD14high CD16+ Monocytes
freq_BM06_mDC (TM2)_in_LIN−
freq_BM06_mDC in pDCs+mDCs
freq_BM06_CD16+ mDC
wbc_BM07_CD4+ CD25++ CCR4+ CCR6− CXCR3− CCR10+
wbc_BM07_CD4+ CD25++ CCR4− CCR6+ CXCR3− CCR10+
wbc_BM07_CD8+ CD25− CCR4− CCR6− CXCR3− CCR10+
freq_BM07_CD8+ CD25− CCR4+ CCR6− CXCR3− CCR10+
wbc_BM07_CD4+ CD25++ CCR4− CCR6− CXCR3+ CCR10+
wbc_BM07_CD4+ CD25++ CCR4+ CCR6− CXCR3+ CCR10+
freq_BM07_CD8+ CD25− CCR4− CCR6+ CXCR3+ CCR10+
freq_BM07_CD8+ CD25− CCR4− CCR6+ CXCR3− CCR10+
wbc_BM07_CD8+ CD25− CCR4− CCR6+ CXCR3+ CCR10+
wbc_BM07_CD8+ CD25− CCR4+ CCR6− CXCR3− CCR10+
wbc_BM07_CD8+ CD25− CCR4− CCR6+ CXCR3− CCR10+
freq_BM07_CD4+ CD25++ CCR4− CCR6+ CXCR3− CCR10+
freq_BM07_CD8+ CD25− CCR4− CCR6− CXCR3− CCR10+
freq_BM07_CD4+ CD25++ CCR4− CCR6− CXCR3+ CCR10+
wbc_BM07_CD4+ CD25++ CCR4− CCR6− CXCR3− CCR10+
freq_BM07_CD4+ CD25++ CCR4− CCR6− CXCR3− CCR10+
freq_BM07_CD8+ CD25− CCR4− CCR6− CXCR3+ CCR10+
wbc_BM07_CD4+ CD25++ CCR4+ CCR6− CXCR3− CCR10−
wbc_BM07_CD4+ CD25++ CCR4− CCR6− CXCR3+ CCR10−
wbc_BM07_CD4+ CD25++ CCR4+ CCR6+ CXCR3− CCR10−
wbc_BM07_CD4+ CD25++ CCR4− CCR6−
wbc_BM07_CD4+ CD25++ CCR4− CCR6− CXCR3− CCR10−
wbc_BM07_CD4+ CD25++ CCR4− CCR6+
wbc_BM07_CD4+ CD25++ CCR4− CCR6+ CXCR3− CCR10−
wbc_BM07_CD4+ CD25++ CCR4− CCR6+ CXCR3+ CCR10+
wbc_BM07_CD4+ CD25++ CCR4− CCR6+ CXCR3+ CCR10−
wbc_BM07_CD4+ CD25++ CCR4+ CCR6−
wbc_BM07_CD4+ CD25++ CCR4+ CCR6− CXCR3+ CCR10−
wbc_BM07_CD4+ CD25++ CCR4+ CCR6+ CXCR3+ CCR10−
wbc_BM07_CD25++CCR4+ (activated/memory Treg)
wbc_BM07_CD4+ CD25++ CCR4+ CCR6+
wbc_BM02_CD3+ Vd2+ CD8+ T cells
wbc_BM02_CD3+ Vd2+ CD4− CD8− T cells
wbc_BM02_CD3+ Vd2+
freq_BM02_CD3+ Vd2+
freq_BM05_class switched memory B cells
freq_BM05_IgD− CD27+
freq_BM07_CD25++CCR4+ (activated/memory Treg)
freq_BM05_IgM+ CD27+
freq_BM05_IgD− IgM+ CD27+ CD38dim
freq_BM05_class non switched memory B cells
freq_BM01_CD3+ T cells
freq_BM07_CD4+ CD25++ CCR4− CCR6− CXCR3+ CCR10−
freq_BM07_CD4+ CD25++ CCR4− CCR6− CXCR3− CCR10−
freq_BM07_CD4+ CD25++ CCR4− CCR6−
freq_BM03_CD4+ CD27+
freq_BM03_CD4+ CD27+ CD28+
freq_BM03_CD4+ CD28+
wbc_BM03_CD4+ CCR7+ CD45RA+
freq_BM03_CD4+ CCR7+ CD45RA+
freq_BM02_TCR ab CD4+ T cells
freq_BM01_CD4+ T cells
wbc_BM07_CD4+ CD25−
wbc_BM02_TCR ab CD4+ T cells
wbc_BM01_CD4+ T cells
wbc_BM03_CD4+ CD28+
wbc_BM03_CD4+ CD27+ CD28+
wbc_BM03_CD4+ CD27+
wbc_BM07_CD4+ CD25−/+ CCR4− CCR6+ CXCR3+CCR10− (pathogenic Th17)
wbc_BM05_class non switched memory B cells
wbc_BM03_CD8 CCR7+ CD45RA+
freq_BM05_CD21+ CD38dim

No rejection & humoral rejection versus cellular rejection

nr_TLR5
freq_BM01_CD14high CD16− Monocytes
wbc_BM06_CD16+ mDC
wbc_BM06_mDC wo mDC1
freq_BM01_CD14+ CD16high Monocytes
freq_BM06_mDC wo mDC1
freq_BM06_mDC (TM2)
freq_BM06_pDCs+mDCs
req_BM06_LIN−HLA−DR+

Suppl. figure 1: Gating strategy chemokine & cytokine panel (panel 7)

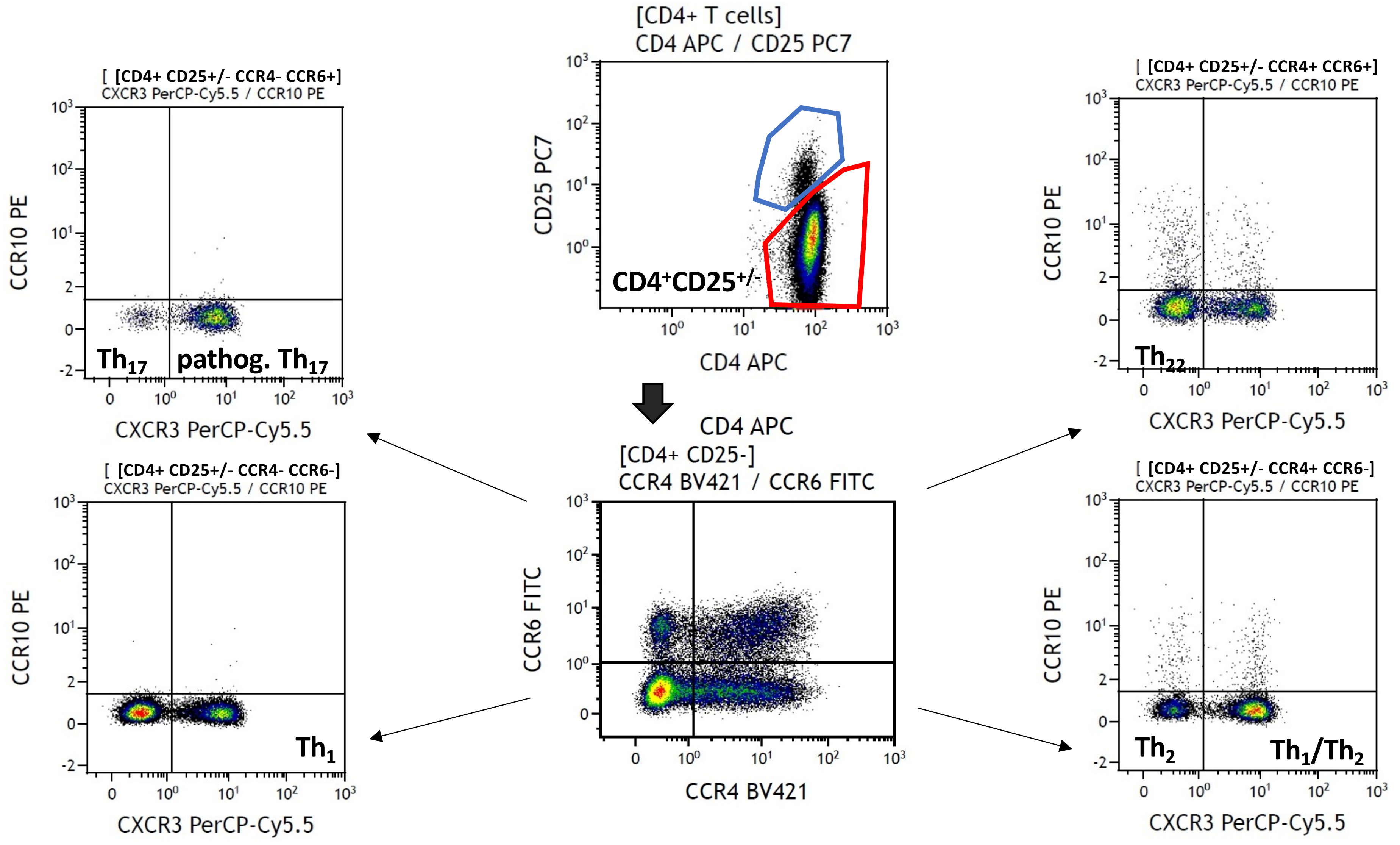

$CD3^+CD4^+$ T cells were subgated into $CD25^{++}$ cells, enriched for regulatory T cells, and $CD25^{-/+}$ conventional T helper cells (upper middle dot plot). Subsequently T cells were further subgated according to CCR4 and CCR6 expression as well as CXCR3 and CCR10 expression distinguishing CCR4- CCR6+ CXCR3- CCR10- Th17-like cells, CCR4- CCR6- CXCR3+ CCR10- Th1-like cells, CCR4- CCR6+ CXCR3+ CCR10- pathogenic Th17-like cells, CCR4+ CCR6+ CXCR3- CCR10- Th22-like cells, CCR4+ CCR6- CXCR3- CCR10- Th2-like cells and CCR4+ CCR6- CXCR3+ CCR10- Th1/Th2-like cells.

Suppl. figure 2: Stability of immune cell subset composition in peripheral blood of ITx patients

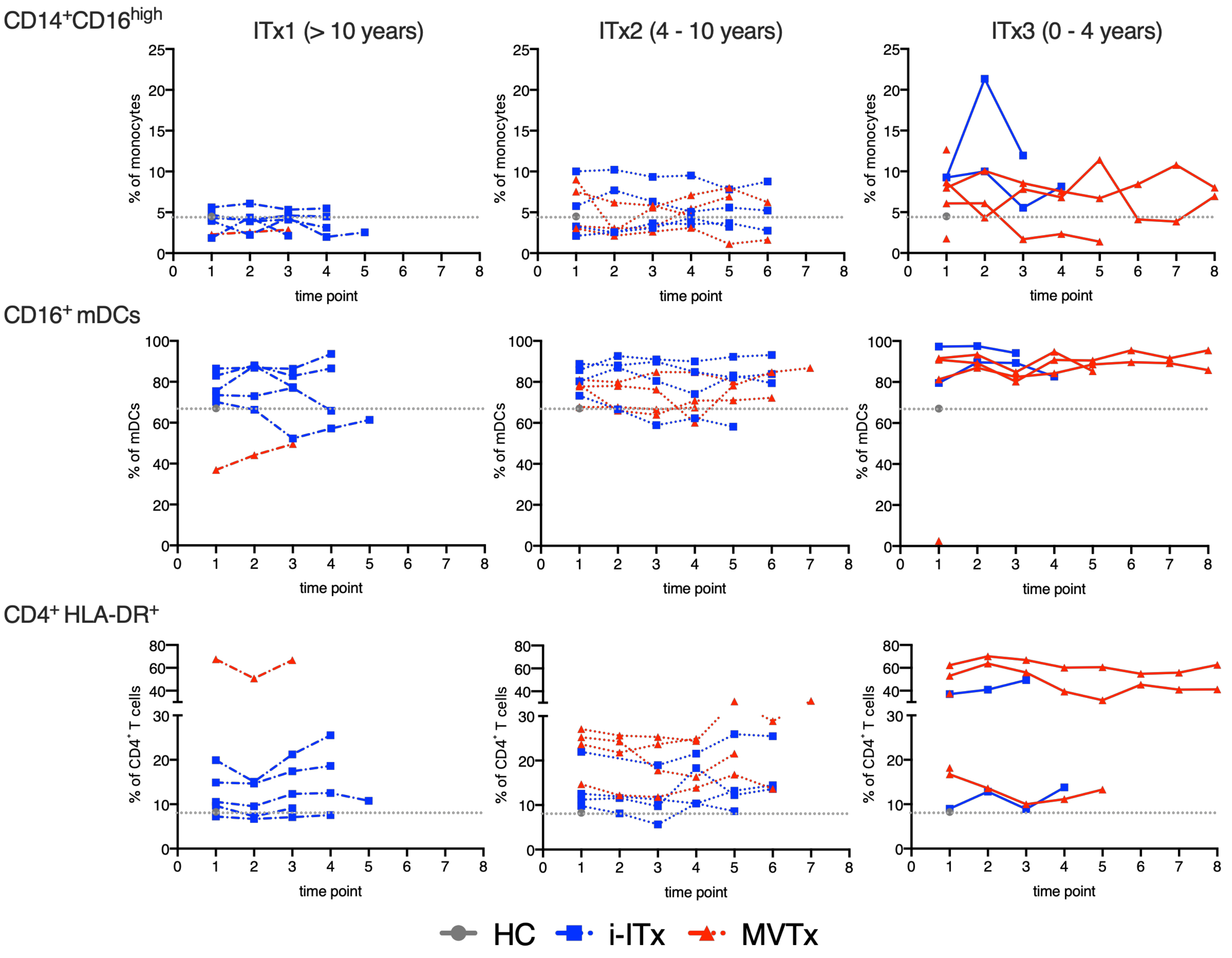

Proportions of indicated innate immune cell subsets in whole blood samples from ITx patients collected one to 8 subsequent time points within one year were analysed by flow cytometry. Results are displayed according to time after transplantation (0-4 years = ITx 3, n=7; 4-10 years = ITx 2, n=8; and >10 years = ITx 1, n=6) post-transplantation and type of transplant (i-Itx = isolated intestinal transplant; MVTx = multivisceral transplant).

Upper row) Proportions of $CD14^{+}CD16^{high}$ monocytes(CV = 24.1%).

Middle row) Proportions of $CD16^{+}$ mDCs (CV = 5.7%).

Bottom row) Proportions of $CD4^{+}HLA\text{-}DR^{+}$ T cells (CV = 14.5%).

Suppl. figure 3: Differences in immune cell subset composition according to organ type

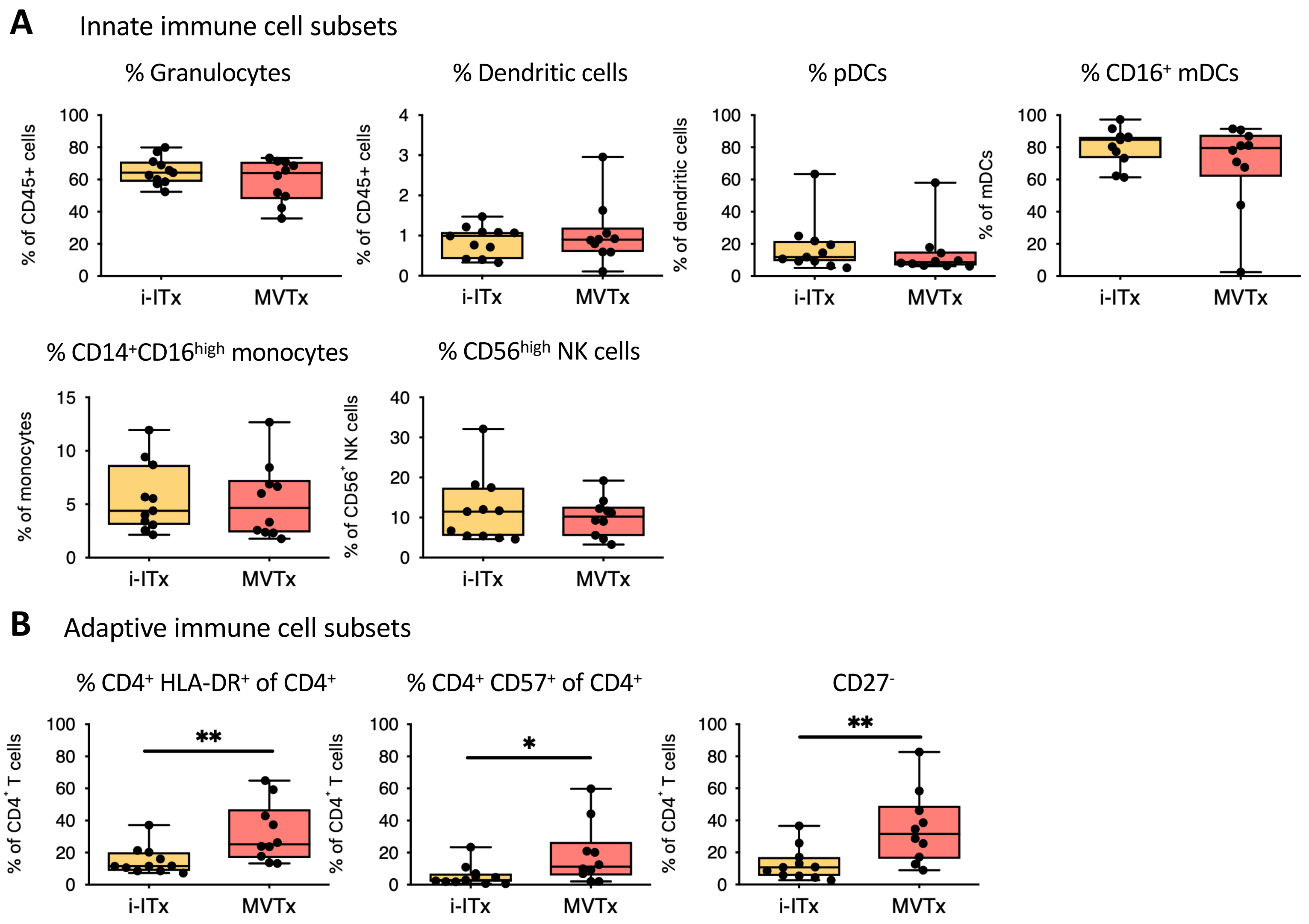

Boxplots showing the median and minimum to maximum of indicated immune cell subsets measured by flow cytometry separating the ITx patients according to type of organ transplant: i-Itx = isolated intestinal transplant; MVTx = multivisceral transplant.

A) Innate immune cells subsets: % of granulocytes of total $CD45^+$ leukocytes, % $LIN^-HLA\text{-}DR^+$ dendritic cells of total $CD45^+$ leukocytes, % $CD11c^-CD123^+$ plasmacytoid dendritic cells (pDCs) of dendritic cells, % $CD14^+CD16^{high}$ monocytes of monocytes, $CD16^+$ myeloid dendritic cells (mDCs), % $CD56^{high}$ of $CD56^+$ NK cells.

B) Activated HLA-DR, CD57 expressing or differentiated $CD27^-$ $CD4^+$ T cells.

Statistical analysis by Kruskal-Wallis-Test and a Conover post-hoc test. * $p<0.05$, ** $p<0.01$

Suppl. figure 4: Gating strategy to enrich cells for chimerism analysis

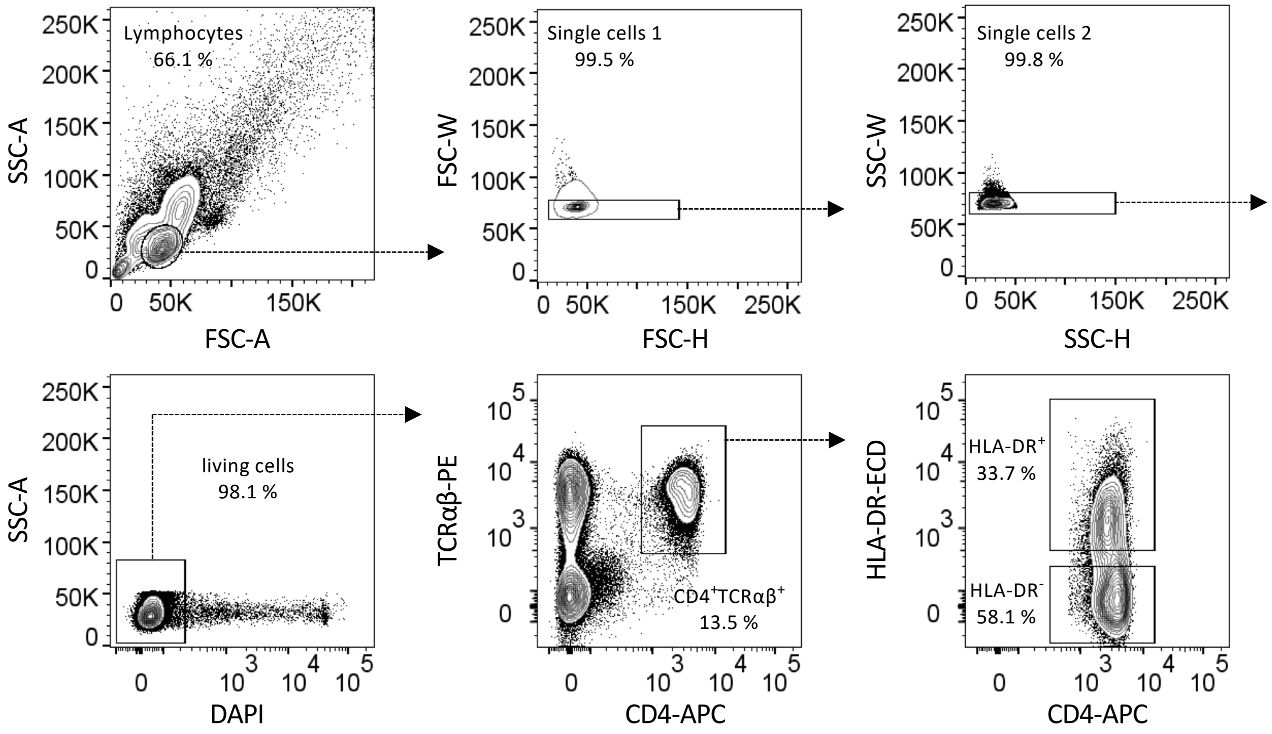

Exemplary gating strategy for FACS-based isolation of HLA-DR⁺ and HLA-DR⁻ CD4⁺ T cells.
Samples were pre-gated on lymphocytes followed by two doublet exclusion gates. Living cells were discriminated from dead cells by viability staining using 4’,6-diamidino-2-phenylindole (DAPI). $CD4^+$ T cells were identified by co-expression of TCRαβ and CD4 and were sorted into HLA-DR^+ and HLA-DR^- fractions.

Suppl. figure 5: TSDR demethylation & *Foxp3* mRNA expression

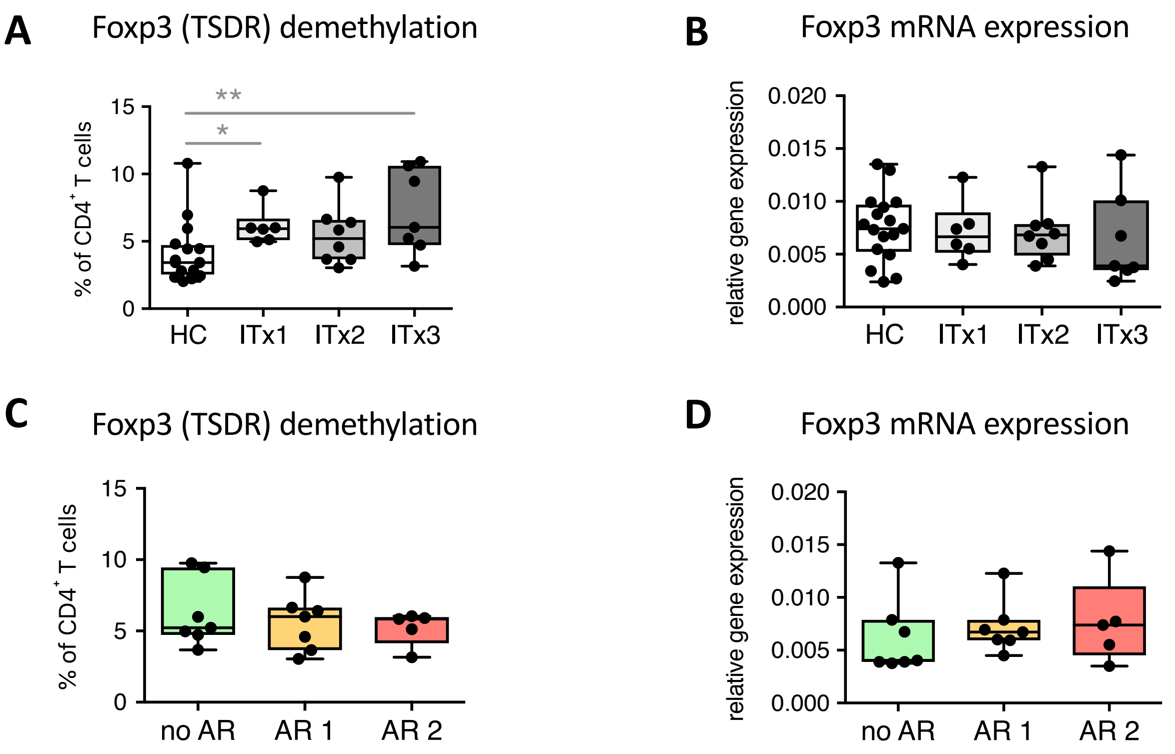

Percentage of CD4+ T cells with demethylation of T reg-specific demethylation region (TSDR, A and C) and *Foxp3* mRNA expression of whole blood samples (B and D) were analysed as described within material and methods. Shown are the results determined according to time after transplantation (A and B, HC: n=, ITx1: n=, ITx2: n=, ITx3: n=) and occurrence of mixed cellular / humoral (AR1) or isolated cellular rejections (C and D, no AR: n=7, AR1: n=7, AR2: n=5). Statistical analysis by Kruskal-Wallis-Test and a Conover post-hoc test. * p<0.05, ** p<0.01

Suppl. figure 6: Serum chemokines & cytokines in association with rejection

A

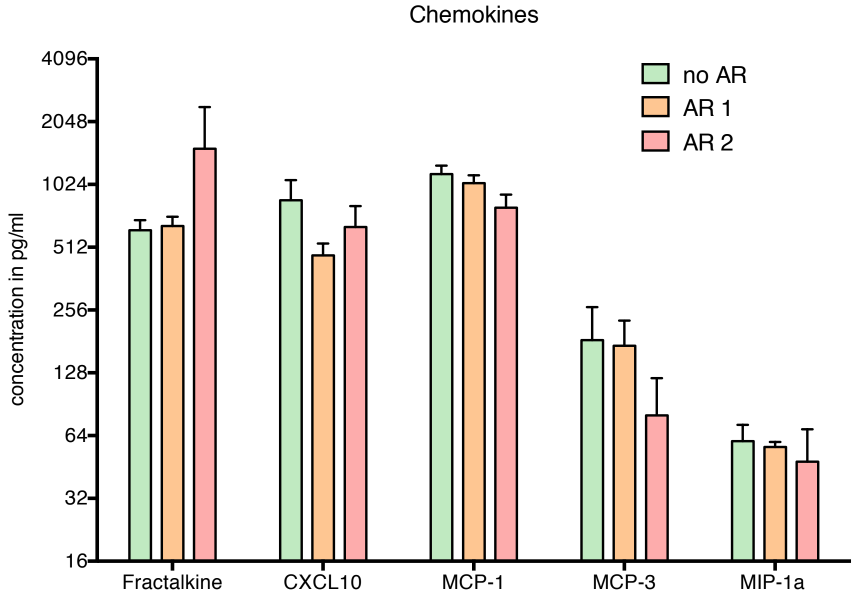

B

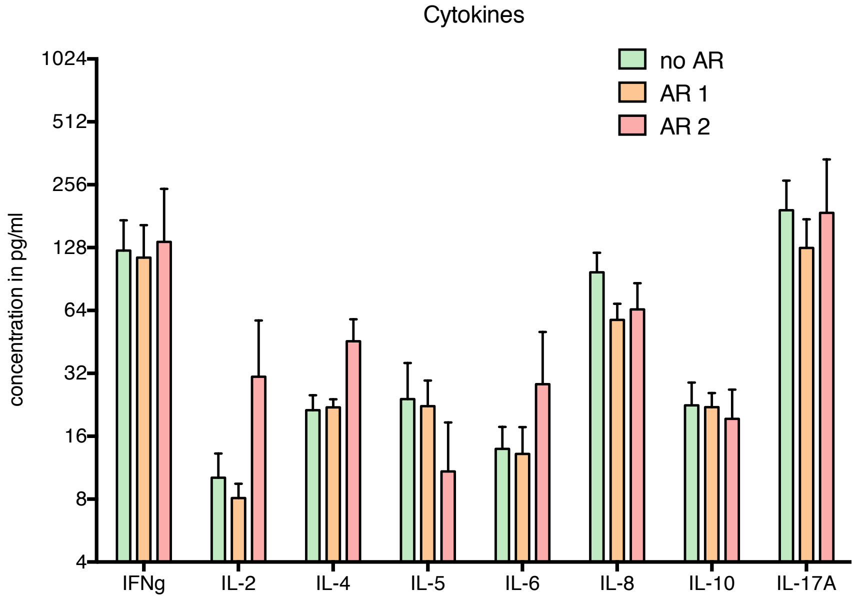

Chemokine (A) and cytokine (B) levels in serum samples of transplant patients (Tx, n=18) were measured using the luminex technology. Transplant patients were categorized according to occurrence of humoral / mixed or cellular rejection (no AR: n=7, AR1: n=7, AR2: n=5).
